# Diagnostic Accuracy of Lung and Abdominal Ultrasound for Tuberculosis in a German Multicenter Cohort of Patients With Presumed Tuberculosis Disease

**DOI:** 10.1093/ofid/ofae651

**Published:** 2024-12-09

**Authors:** Stefan Fabian Weber, Peter Wolf, Nils Wetzstein, Claus Küpper-Tetzel, Maria Vehreschild, Isabelle Suárez, Jan Rybniker, Angela Klingmüller, Tim Weber, Maximilian Güttlein, Frank Tobian, Lisa Koeppel, Julia Selena Beck, Rebecca Wolf, Katharina Manten, Stefan Zimmermann, Devasahayam Jesudas Christopher, Felix Herth, Sabine Bélard, Claudia M Denkinger, For the ALL POCUS TB Germany Study group, Lisa C Ruby, Mary Gaeddert, Michael Weise, Monika Eichinger, Benjamin Blöck, Fabian Sattaf, Anna-Lia Prey, Alena Drummer, Dominic Rauschning, Daniel Ludwig-Bettin, Elisa Pracht

**Affiliations:** Department for Infectious Disease and Tropical Medicine, University Hospital Heidelberg, Heidelberg, Germany; Department for Parasitology, University Hospital Heidelberg, Heidelberg, Germany; German Center for Infectious Disease Research, DZIF Partner Site Heidelberg, Heidelberg, Germany; Department for Pneumology and Critical Care Medicine, Thoraxklinik Heidelberg, Heidelberg, Germany; Department 2 of Internal Medicine, Infectious Diseases, University Hospital Frankfurt, Goethe University Frankfurt, Frankfurt am Main, Germany; Department 2 of Internal Medicine, Infectious Diseases, University Hospital Frankfurt, Goethe University Frankfurt, Frankfurt am Main, Germany; Department 2 of Internal Medicine, Infectious Diseases, University Hospital Frankfurt, Goethe University Frankfurt, Frankfurt am Main, Germany; Department I of Internal Medicine, Division of Infectious Diseases, Medical Faculty and University Hospital of Cologne, University of Cologne, Cologne Germany; German Center for Infectious Disease Research, DZIF Partner Site Cologne, Cologne, Germany; Department I of Internal Medicine, Division of Infectious Diseases, Medical Faculty and University Hospital of Cologne, University of Cologne, Cologne Germany; German Center for Infectious Disease Research, DZIF Partner Site Cologne, Cologne, Germany; Center for Molecular Medicine Cologne, University of Cologne, Cologne, Germany; Department I of Internal Medicine, Division of Infectious Diseases, Medical Faculty and University Hospital of Cologne, University of Cologne, Cologne Germany; German Center for Infectious Disease Research, DZIF Partner Site Cologne, Cologne, Germany; Department of Diagnostic and Interventional Radiology, University Hospital Heidelberg, Heidelberg, Germany; Department of Diagnostic and Interventional Radiology, University Hospital Heidelberg, Heidelberg, Germany; Department for Infectious Disease and Tropical Medicine, University Hospital Heidelberg, Heidelberg, Germany; German Center for Infectious Disease Research, DZIF Partner Site Heidelberg, Heidelberg, Germany; Department for Infectious Disease and Tropical Medicine, University Hospital Heidelberg, Heidelberg, Germany; Department for Infectious Disease and Tropical Medicine, University Hospital Heidelberg, Heidelberg, Germany; Department for Infectious Disease and Tropical Medicine, University Hospital Heidelberg, Heidelberg, Germany; German Center for Infectious Disease Research, DZIF Partner Site Heidelberg, Heidelberg, Germany; German Center for Infectious Disease Research, DZIF Partner Site Heidelberg, Heidelberg, Germany; Department of Anaesthesiology, University Hospital Heidelberg, Heidelberg, Germany; Department for Infectious Diseases, University Hospital Heidelberg, Institute of Medical Microbiology and Hygiene, Heidelberg, Germany; Department of Pulmonary Medicine, Christian Medical College Vellore-Ranipet campus, Vellore, India; Department for Pneumology and Critical Care Medicine, Thoraxklinik Heidelberg, Heidelberg, Germany; Translational Lung Research Center Heidelberg, Heidelberg, Germany; University of Tübingen, Institute of Tropical Medicine, Tübingen, Germany; German Center for Infectious Disease Research, DZIF Partner Site Tübingen, Tübingen, Germany; Department for Infectious Disease and Tropical Medicine, University Hospital Heidelberg, Heidelberg, Germany; German Center for Infectious Disease Research, DZIF Partner Site Heidelberg, Heidelberg, Germany

**Keywords:** diagnosis, lung ultrasound, migrant medicine, point-of-care ultrasound, tuberculosis

## Abstract

**Background:**

There is limited evidence on point-of-care ultrasound for tuberculosis (TB), but studies suggest high sensitivity, especially for lung ultrasound (LUS). However, insufficient data are available on specificity of the examination and its generalizability to a broader patient population.

**Aims:**

Our study aimed to establish accuracy for lung, chest, and abdominal ultrasound, individually and in combination, for TB diagnosis.

**Methods:**

We conducted a prospective diagnostic accuracy study among consecutive adult out- and inpatients with probable TB in three German referral hospitals. We applied a comprehensive standardized ultrasound protocol. TB diagnosis was established by a microbiological reference standard including polymerase chain reaction and culture.

**Results:**

A total of 102 participants originating from 30 different countries were enrolled. HIV prevalence was 7/99 (7%) and 73/102 (72%) had confirmed TB. TB was limited to the lungs in 15/34 (44%) of refugees and 27/39 (69%) in nonrefugees. Focused assessment with sonography for HIV-associated tuberculosis had a sensitivity of 40% (95% confidence interval [CI], 30–52) and specificity of 55% (95% CI, 38–72). Additional findings, such as small subpleural consolidations on LUS had a high sensitivity (88%; 95% CI, 78–93), but a low specificity (17%; 95% CI, 8–35). Larger consolidations in the lung apices had a sensitivity of 19% (95% CI, 12–30) and a specificity of 97% (95% CI, 83–100).

**Conclusions:**

Our study establishes the first data on LUS performance against a comprehensive reference standard. Overall, our data suggest that ultrasound does not meet the requirements for triage but previously described and novel ultrasound targets in combination could aid in the clinical decision making.

Registry: DRKS00026636

## Teaser

This German multicenter study prospectively investigated lung and abdominal point-of-care ultrasound in patients with presumed tuberculosis and revealed limited accuracy. Point-of-care ultrasound has limited value as a standalone test but could be part of an algorithmic approach for diagnosis.

In 2022, tuberculosis (TB) incidence was 10.6 million globally, with pulmonary TB (PTB) constituting 83% of cases globally and 76% of cases in Germany. The global case detection gap in the same year was 26% [1, 2]. The World Health Organization (WHO) published target product profiles for diagnostic triage tests for TB requiring a minimum sensitivity of 90% and specificity of 70% [3]. Chest x-ray (CXR) is the most widely used triage test to date to direct symptomatic, healthcare-seeking patients to appropriate further confirmatory testing. Its scalability is improved by computer assisted detection [4] and decentralized instruments (ultraportable CXR systems [5]); however, it remains too costly or unavailable at many peripheral settings in many TB-endemic countries because of the infrastructure needs.

Point-of-care ultrasound (POCUS) for TB has emerged as a portable low-cost alternative. The focused assessment with sonography for HIV-associated tuberculosis (FASH) protocol targeted those with HIV and assesses for pleural and pericardial effusion of any size, abdominal lymphadenopathy, as well as splenic and liver microabscesses [6–8]. In previous studies however, study design (control group and target population) has often limited generalizability of the findings. The lack of generalizable data is especially relevant outside settings the FASH protocol was originally developed to address (ie, populations without HIV). Previous studies showed sensitivities ranging from 36% to 39% and specificities between 70% and 89% [9–11].

Lung ultrasound (LUS) for TB has recently drawn attention, but its role for point-of-care applications remains unclear. Studies suggested “subpleural nodules” and a “miliary pattern” to be associated with PTB (eg, [12, 13]). A systematic review suggested that sensitivity for subpleural nodules exceeds 90%, but specificity remains unclear because of choice or lack of adequate control groups and lack of clear definitions [14]. Findings like lymphadenopathy in the internal mammary region [15, 16], mediastinal lymph nodes in adult patients [17], or peritoneal TB [18] have not been evaluated in a prospective study.

In this study, we aimed to investigate the accuracy of the FASH protocol as well as LUS and novel ultrasound targets for a point-of-care use case in a triage setting of symptomatic patients with presumed TB in a high-resource tertiary care hospital setting in Germany, applying a comprehensive workup to arrive at definite diagnosis (ie, TB or other).

## METHODS

### Study Design and Participants

ALL POCUS TB (abdominal and lung POCUS for TB) was a multicenter, prospective diagnostic accuracy study at three German referral hospitals: University Hospitals Heidelberg, Frankfurt, and Cologne. A parallel study in an Indian referral hospital used similar methods but had substantial differences in participant populations. Results are published separately [19].

Study staff reviewed medical charts of in- and outpatients for inclusion criteria. We included consecutive adults aged ≥18 years with presumed or recently confirmed pulmonary or extrapulmonary TB disease who were positive for WHO TB 4-symptom screen [20], representing the use case as encountered in clinical care for triage/screening and acknowledging preselection. We excluded patients if TB was diagnosed by microbiological means (polymerase chain reaction [PCR]; culture) > 14 days before screening; OR, if anti-TB treatment had been taken >7 days; OR, if any TB-active medication had been taken in the past 6 months, to exclude impact on pretreatment on ultrasound findings.

The study was approved by respective ethics committees (Heidelberg S-314/2021; Frankfurt 2021-466; Cologne 22-1009). The study was conducted according to Good Clinical Practice and the Helsinki declaration. Written informed consent was obtained from all participants. The study was registered with the German clinical trial registry (DRKS00026636). This study conforms to the Standards for Reporting of Diagnostic Accuracy Studies reporting guidelines, see Supplement [21]. All authors declare no conflict of interest.

### Procedures

Demographic data and medical history of eligible and consenting participants were recorded in standardized clinical report forms. Refugee status was defined by self-declaration. Routine tests included HIV serology (positive status if positive current test or self-reported prior positive test), HbA1c (diabetes positive if HbA1c of ≥6.5% or self-reported prior diagnosis of diabetes), and C-reactive protein (CRP).

TB tests included at least 2 respiratory samples (sputum or bronchoalveolar lavage) with liquid culture and at least 1 PCR test. Other investigations and imaging were performed as indicated by the attending physician. Relevant data were extracted from hospital records. We contacted all participants after 2 months for a clinical follow-up (in person or via telephone). Participants with negative TB tests not empirically treated for TB, but experiencing persistent symptoms, were offered sputum retesting after 2 months.

### Index Test

FASH: The study POCUS protocol included FASH_original_ as published [6]. In addition to the predefined protocol, we added pericardial (FASH_pericardium_), ascites (FASH_ascites_), and pleural effusion (FASH_pleural_) measurements (adapted from Goecke et. al. [22]) to assess effects of altered cutoffs on accuracy (definitions, see Table 1).

**Table 1.** Definitions of Ultrasound Findings and FASH Variations

	Definition	Further Characteristics Assessed	Location
SPC	Subpleural consolidations, hypoechoic or mixed echoic lesions originating from the visceral pleura	max. vertical dimension or translobar if not measurable, bronchograms	14 lung zones
B-lines	Vertical lines originating from visceral pleura and extending to >50% of view field, minimum of 3 per lung zone	-	14 lung zones
Miliary pattern	B-lines, comet artefacts, pleural irregularities, small subpleural consolidations affecting all lung fields	-	14 lung zones
Pleural thickening	Thickening of parietal or peritoneal pleura	Laminar thickening or nodular thickening, maximum thickness	14 lung zones
Pleural fluid	Content of hypoechoic or mixed echoic echogenicity between parietal and visceral pleura	Echogenicity, measurement of basal lung to diaphragm distance and maximum craniocaudal distance, estimation of volume (22)	Bilateral pleural recessus
Peritoneal thickening	Thickening of the peritoneal lining in the parietal, visceral, or omental layers	Maximum thickness, inclusion of hypoechoic lesions, laminar or nodular thickening	4 abdominal quadrants, transcostal liver view
Intestinal thickening	Thickening of the intestinal wall in the terminal ileum region exceeding 4 mm	-	Right lower abdominal quadrant
Peritoneal fluid	Free abdominal fluid	Amount (small, moderate, large), echogenicity, content	Hepatorenal, splenorenal, and retrovesical pouch as well as inter-intestinal
Pericardial fluid	Pericardial fluid ≥4 mm	Measurement, echogenicity, content	Subxiphoidal view
Spleen lesions	Intra-parenchymal spleen lesions Hypoechoic <1.5 cm Hypoechoic ≥1.5 cm echogenic	Measurement, count	Left flank
Liver lesions	Intra-parenchymal hypoechoic lesions	Measurement, count	Subcostal, transcostal
Abdominal lymph nodes	Lymph nodes ≥1.5 cm any dimension	Measurement, architecture, bulking	Liver and spleen hilum, peripancreatic, para-aortic
Internal mammary lymph nodes	Lymph nodes ≥0.5 cm, any dimension	Measurement, side, architecture, bulking	Parasternal intercostal spaces
Mediastinal lymph nodes	Lymph nodes ≥1.5 cm, any dimension	Measurement, side, architecture, bulking	Suprasternal view, parasternal view
FASH_original_	Abdominal lymph nodes ≥1.5 cm Hypoechoic liver or spleen lesions Pleural effusion, any amount Pericardial effusion ≥1cm		
FASH_ascites_	Abdominal lymph nodes ≥1.5 cm Hypoechoic liver or spleen lesions Pleural effusion, any amount Pericardial effusion ≥1 cm Ascites, any amount		
FASH_pericardium_	Abdominal lymph nodes ≥1.5 cm Hypoechoic liver or spleen lesions Pleural effusion, any amount Pericardial effusion ≥0.4 cm		
FASH_pleural_	Abdominal lymph nodes ≥1.5 cm Hypoechoic liver or spleen lesions Pleural effusion, minimum amount specified Pericardial effusion ≥1 cm		

Abbreviations: FASH, focused assessment with sonography for HIV-associated tuberculosis; SPC, subpleural consolidations.

Underlined aspects highlight differences to original FASH protocol.

**Figure 1.**
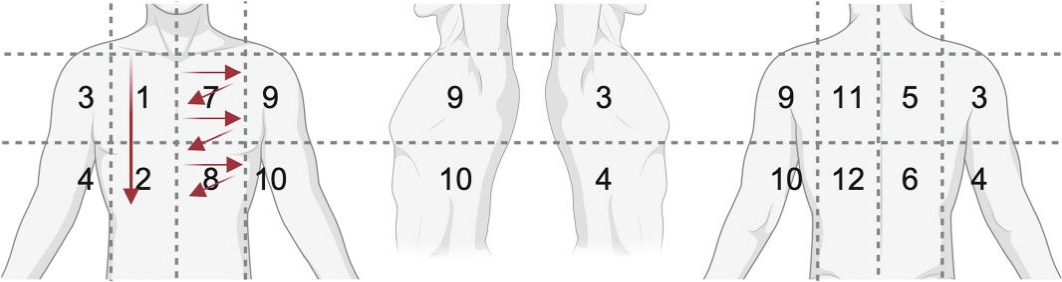
schematic of lung zones and probe movement. Zone 1/2: example for longitudinal sweep; zone 7/8: example for intercostal sweeps.

LUS: We adapted existing lung protocols for 14 lung zones (Figure 1) [23]. All zones were scanned vertically and parallel to the intercostal spaces with a linear probe, with optional changing to curved probe (eg, in case of poor visualization). We documented presence of A-lines, B-lines, pleural effusions, subpleural consolidations (SPCs), and miliary pattern. In addition to these predefined findings, we explored the accuracy of different numbers, sizes, and locations of findings.

Other targets: we assessed for internal mammary lymph nodes (IMNs) in the parasternal intercostal spaces, peritoneal or omental thickening, and ileocecal thickening. Using a phased array probe, we attempted to visualize mediastinal lymphadenopathy from suprasternal notch and left parasternal view. In participants with presumed peripheral TB lymphadenopathy, we scanned respective areas.

In Heidelberg, ultrasound was performed on the day of enrollment by a nonradiologist clinician (S.F.W., 8 years of TB-POCUS experience). In Frankfurt and Cologne, nonradiologist clinicians with ultrasound experience ranging between basic and advanced skills were trained by S.F.W. with proficiency ensured by trial clips evaluated for protocol adherence. Ultrasound investigators were aware of participants’ TB diagnosis if known at time of inclusion, but unaware of imaging results. Ultrasound clips were recorded and interpreted in a standardized clinical report forms by S.F.W.; inadequately visualized views were marked nonevaluable. For ultrasound rater agreement, a random subset of 15% of participants from all study sites were evaluated by an additional blinded rater independently (S.F.W., R.W., K.M.) regarding SPC _≥ 1cm_, SPC _< 1cm_, and FASH. We used an Edge II (Fujifilm Sonosite, Japan) in Heidelberg, provided as an in-kind loan from Fujifilm. In Frankfurt, we used Aplio 300 (Toshiba, Japan) and Acuson Juniper (Siemens, Germany) and in Cologne we used HS40 (Samsung, South Korea).

### Comparator Test

For a subset (59/83, 71%) of available participants, CXRs (performed within 6 weeks of recruitment), were assessed by 2 radiologists blinded to clinical and index test data (consensus read). Radiologists interpreted images as “CXR suggesting likely TB” or “CXR suggesting possible TB”, “suggestive of post-TB sequelae” (with/without signs of active TB) or “not suggestive of active TB or post-TB sequelae” [24]. CRP as a predictor was evaluated at a cutoff of 5 mg/L [20]. CXR and CRP were not included in the reference standard.

### Reference Standard and Case Definitions

Participants with at least 1 respiratory sample examined with TB-culture or at least 2 respiratory samples tested with TB-PCR were included in the analysis. For participants with presumed exclusive extrapulmonary TB (EPTB), no respiratory sample was required.

Predefined criteria for TB were (1) a microbiological reference standard (MRS, any respiratory sample positive by culture or PCR); (2) an extended MRS (eMRS, any other sample positive on culture or PCR); and (3) a not predefined composite reference standard (CRS, empirical anti-TB treatment started with clinical improvement at follow-up). TB was considered unlikely if MRS, eMRS, and CRS were negative AND 1 of the following criterion was met: follow-up with negative sputum, or symptom resolution, or a plausible differential diagnosis (predefined, see Statistical Analysis Plan in Supplement). All other cases were assessed by an outcome committee (2 senior TB physicians [C.M.D., P.W.]) blinded to the index test with access to relevant clinical data. Cases were assigned positive CRS, unlikely TB, or unclassifiable status by consensus decision (excluded from analyses).

### Statistics

Target sample size for participants was n = 220 and TB cases n = 44 detected with index test (inputs: sensitivity 60%, specificity 85%, disease prevalence 52.5%, loss to follow-up 20%) [25]. Standardized data collection was done using RedCap (Version R4.2.2) [26]. Missing data are indicated by denominators or footnotes. Our primary reference standard for accuracy calculation was CRS. Sensitivity and specificity were calculated with 95% confidence intervals (95% CI). Subgroup analyses were conducted for HIV and diabetes. For rater agreement, we used Cohen's kappa and a generalized linear mixed model for the probability of agreement. Analyses were done in R (v.4.2.2, packages: openxlsx, REDCapR, ggplot2, dplyr, Hmisc). Figures were created with biorender.com and R.

## RESULTS

**Figure 2.**
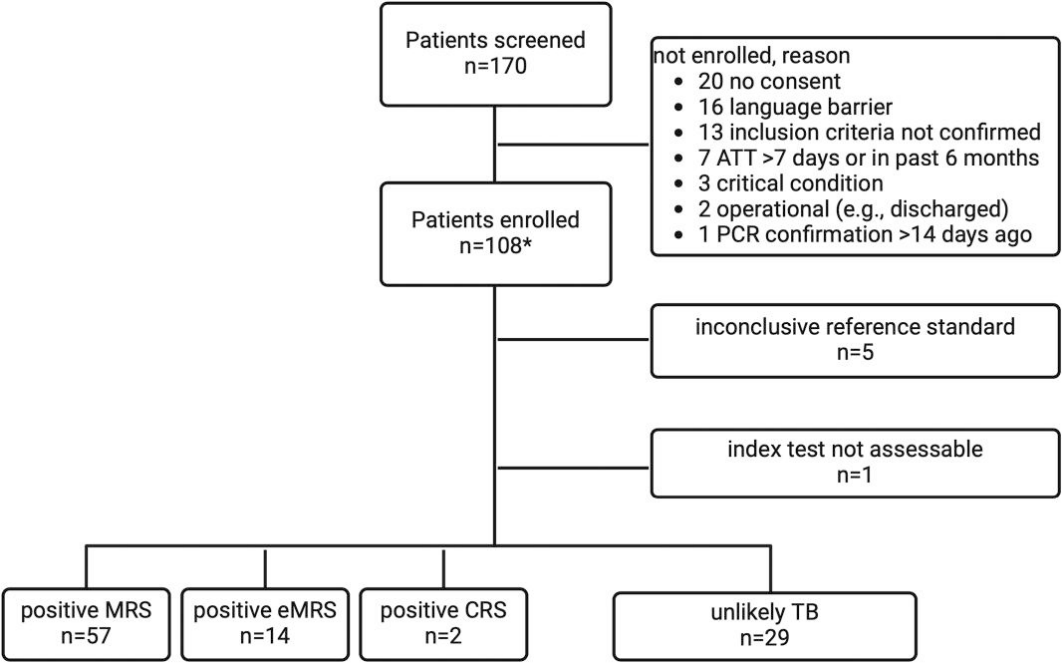
Flow chart for study recruitment and reference standard categories. Abbreviations: CRS, composite reference standard; eMRS extended MRS; MRS, microbiological reference standard; TB, tuberculosis; UKC, University Hospital Cologne; UKF, University Hospital Frankfurt; UKHD, University Hospital Heidelberg. *UKHD n = 83, UKF n = 19, UKC n = 6.

Between 20 January 2022 and 26 July 2023, we screened 170 individuals and enrolled 108 participants. One participant was excluded because of insufficient samples, 5/108 (5%) were unclassifiable because of loss of follow-up, and we included 102/108 (94%) in the analysis. In 1 participant, the index test (ultrasound) could not be assessed for technical reasons (Figure 2).

### Study Population

Median age was 40 years and 80/102 (78%) were male. Of 102 participants, 17 (17%) were of German origin, the remainder were from 30 other countries (Table 2, footnote). The majority (53/102, 52%) self-identified as refugees. Diabetes and HIV prevalence were 13/69 (19%) and 7/99 (7%), respectively. Participant characteristics are provided in Table 2, and MRS/eMRS data in Supplementary Table 1.

**Table 2.** Participant Characteristics and Reference Standard Testing

Variables In N, Median, IQR, (%)		All Participants (n = 102)	PTB Cases (MRS Positive) (n = 57)	TB Cases (CRS Positive) (n = 73^a^)	Unlikely TB (n = 29)
Age in years		40 [30;47] (N = 102)	41 [32;47] (N = 71)	41 [31;47] (N = 73)	39 [30;43] (N = 29)
Gender male		80/102 (78%)	55/71 (77%)	55/73 (75%)	25/29 (86%)
Origin as per WHO regions^b^		43/102 (42%) European Region 25/102 (25%) African Region 23/102 (23%) Eastern Mediterranean Region 9/102 (9%) South-East Asian Region 2/102 (2%) Western Pacific Region	…	…	…
Identifies as refugee		53/102 (52%)^c^	26/57 (46%)	34/73 (47%)	19/29 (66%)
Diabetes		13/69 (19%)	4/52 (8%)	7/45 (16%)	6/24 (25%)
HIV status		7/99 (7%)	4/55 (7%)	5/71 (7%)	2/28 (7%)
History of previous TB disease		9/100 (9%)	6/56 (11%)	6/72 (8%)	3/28 (11%)
History of TB-contact		29/100 (29%)	16/56 (29%)	22/72 (31%)	7/28 (25%)
Symptoms					
Cough		80/102 (78%)	45/57 (79%)	53/73 (73%)	27/29 (93%)
Night sweats		45/102 (44%)	27/57 (47%)	33/73 (45%)	12/29 (41%)
Fever		32/98 (33%)	19/54 (35%)	22/70 (31%)	10/28 (36%)
Weight loss		60/99 (61%)	37/54 (69%)	46/70 (66%)	14/29 (48%)
Abdominal pain or distension		13/100 (13%)	4/55 (7%)	9/71 (13%)	4/29 (14%)
Test results					
C-reactive protein (mg/L) > 5 mg/L^d^		63/102 (62%)	36/57 (63%)	46/73 (63%)	17/29 (59%)
CXR suggesting likely TB		11/59 (19%)	10/28 (36%)	10/37 (27%)	1/22 (5%)
CXR suggesting possible TB (includes likely)		36/59 (61%)	24/28 (86%)	25/37 (68%)	11/22 (50%)
Number of sputa investigated for TB (≥2; < 2 samples)		98/102 (96%); 4/102 (4%)	56/57 (98%); 1/57 (2%)	69/73 (95%); 4/73 (5%)	29/29 (100%); NA
Sputum smear status (% where done)^e^	Negative	60/89 (67)	19/48 (40)	31/60 (52)	29/29 (100)
	Scanty	1/89 (1)	1/48 (2)	1/60 (2)	NA
	1+	6/89 (7)	6/48 (13)	6/60 (10)	NA
	2+	6/89 (7)	6/48 (13)	6/60 (10)	NA
	3+	16/89 (18)	16/48 (33)	16/60 (27)	NA
	Not done	10	7	10	NA
Positive TB-PCR or culture on sputum or BAL		57/102 (56%)	57/57 (100%)	57/73 (78%)	0/29 (0%)
Positive TB-PCR or culture on non-sputum/non-BAL sample		32/102 (31%)	18/57 (32%)	32/73 (44%)	0/29 (0%)

Denominators provided for all individuals with available data for each line.

Abbreviations: ART, antiretroviral therapy; BAL, broncho-alveolar lavage; CRS, composite reference standard; CXR, chest x-ray; eMRS, extended MRS; EPTB, extrapulmonary tuberculosis; IQR, interquartile range; m, month(s); MRS, microbiological reference standard; PCR, polymerase chain reaction; TB, tuberculoisis; WHO, World Health Organization; y, year(s).

^a^42/73 (58) PTB only; 14/73 (19) EPTB only; 17/73 (23) PTB + EPTB; the most common EPTB locations were peripheral lymphadenopathy (n = 9), abdominal lymphadenopathy (n = 6), pleura (n = 5).

^b^Countries of origin: Germany: 17; Eritrea: 9; Romania: 8; Ukraine: 8; India: 7; Somalia: 7; Gambia: 5; Pakistan: 5; Syria: 4; Afghanistan: 3; Morocco: 3; Algeria: 2; Georgia: 2; Kazakhstan: 2; Senegal: 2; Sudan: 2; Turkey: 2; Vietnam: 2; Bangladesh: 1; Cameroon: 1; Ethiopia: 1; France: 1; Jordan: 1; Kenya: 1; Moldavia: 1; Poland: 1; Sierra Leone: 1; Slovakia: 1; Thailand: 1; Togo: 1.

^c^ < 1 m: 13/53 (25%); 1–12 m: 17/53 (32%); 1–5 y: 6/53 (11%); > 5 y: 17/53 (32%).

^d^Sensitivity 63% (95% confidence interval 52–73%), specificity 41% (95% confidence interval 26–59).

^e^Definition, see Global Laboratory Initiative, Laboratory diagnosis of tuberculosis by sputum microscopy. 2013: South Australia.

**Figure 3.**
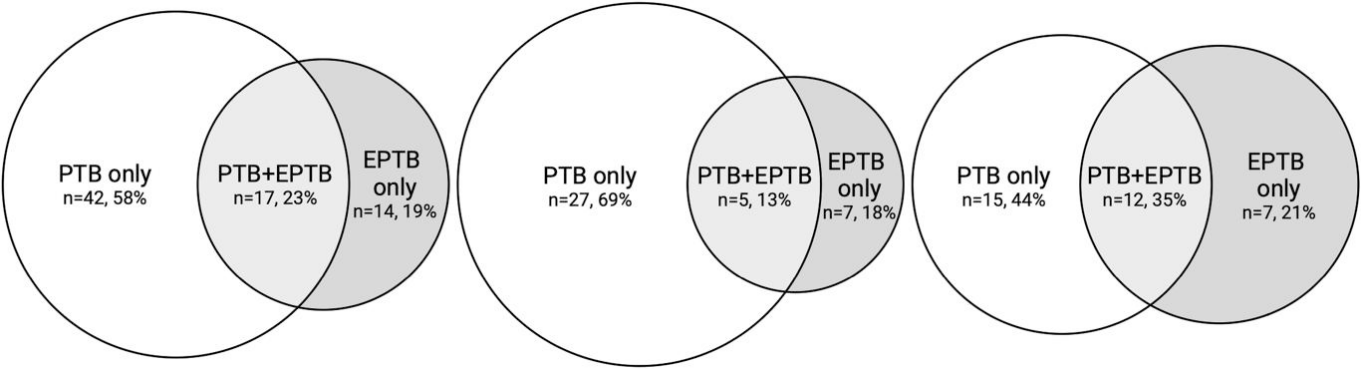
Tuberculosis location and refugee status. Venn diagrams for location of TB in all participants, self-identified refugees and nonrefugees. *A* (left), all TB cases. *B* (center), nonrefugee TB cases. *C* (right), refugee TB cases. Abbreviations: EPTB, extrapulmonary tuberculosis; PTB, pulmonary tuberculosis; PTB + EPTB: concurrent PTB and EPTB; TB, tuberculosis.

TB was diagnosed in 73/102 (72%), with 57/73 (78%) identified as per MRS+, 14/73 (19%) as per eMRS+ and 2/73 (3%) in addition as per only CRS+ . Of those, 42/73 (58%) cases had PTB only, 14/73 (19%) had EPTB without PTB, 17/73 (23%) had concurrent PTB + EPTB (Figure 3*A*), and 29/102 (28%) had unlikely TB. TB cases in refugees were less often PTB alone (15/34, 44%) and more frequently EPTB (solitary EPTB: 7/34, 21%; concurrent PTB + EPTB 12/34, 35%) in comparison with nonrefugee participants (PTB: 27/39, 69%; EPTB 7/39, 18%; concurrent PTB + EPTB 5/39, 13%), see Figure 3*B* and 3*C*.

For participants with unlikely TB, the most common alternative diagnoses were non-TB lung infections (6/29, 21%) and post-TB sequelae (4/29, 14%) (Table 3).

**Table 3.** Differential Diagnoses for Participants With Unlikely Tuberculosis

	N	Non-TB Lung Infection	Post-TB Sequelae	ILD	Systemic Infections	Asthma	COPD OR Other Obstructive Lung Disease	Lung Neoplasia	Hematological Malignancy	Chronic Kidney Disease	Bronchiectasis
All unlikely TB	29	6	4	2	2	1	1	1	1	1	1
FASH_original_	13	2	1	0	2	1	1	1	0	1	1
Pleural effusion, any	11	2	1	0	2	1	1	1	0	1	0
Pleural effusion >400 mL	3	0	1	0	0	1	0	0	0	1	0
Pericardial effusion >1 cm	2	0	1	0	0	0	0	0	0	0	1
Spleen lesions	3	0	1	0	1	0	0	0	0	0	0
Liver lesions	2	0	0	0	1	0	0	1	0	0	0
Abdominal lymph nodes	1	0	0	0	1	0	0	0	0	0	0
Ascites	2	0	1	0	0	0	0	0	0	1	0
Internal mammary lymph nodes	3	1	1	0	0	0	0	0	0	0	0
Peritoneal thickening	0	0	0	0	0	0	0	0	0	0	0
SPC _< 1cm_	24	6	4	2	2	1	1	1	0	1	1
SPC _≥ 1cm_	13	3	3	0	2	1	0	1	0	1	0
Miliary pattern	1	0	0	0	0	0	0	0	0	0	1
CXR suggesting likely TB	0	0	0	0	0	0	0	0	0	0	0

Abbreviations: COPD, chronic obstructive pulmonary disease; CXR, chest x-ray; FASH, focused assessment with sonography for HIV-associated tuberculosis; ILD, interstitial lung disease; SPC, subpleural consolidations; TB, tuberculosis.

### FASH

FASH_original_ had a sensitivity of 40% and specificity of 55%. The most common FASH findings were pleural effusion (23/101, 32%) and abdominal lymphadenopathy (9/101, 9%). Pericardial effusion (5/101, 5%), splenic (4/101, 4%), and liver lesions (2/101, 2%) were rare. The addition of a pleural effusion cutoff of 600 mL (exemplary) optimized specificity to 79% (95% CI, 62–92) but reduced sensitivity to 22%. Lowering the pericardial effusion cutoff to 4 mm or adding ascites into FASH did not change FASH accuracy relevantly. For details, see Table 4. For results applying further variations to FASH, see Supplementary Table 2. Limiting TB cases to those with EPTB (n = 31) did not improve FASH_original_ sensitivity (12/31 [39%], Table 4). In unlikely TB, FASH findings were found in a variety of alternative diagnoses (Table 3).

**Table 4.** Abdominal and Lung Ultrasound Findings and Variations

Variables in N, Median, IQR, (%)	All Participants (n = 102)	PTB Cases (MRS Positive) (n = 57)	TB Cases (CRS Positive) (n = 73)	Unlikely TB (n = 29)	Sensitivity (95% CI)	Specificity (95% CI)
FASH						
FASH_original_^a^	42/101 (42%)	25/56 (45%)	29/72 (40%)	13/29 (45%)	0.4 [0.3–0.52]	0.55 [0.38–0.72]
FASH_ascites_	44/101 (44%)	27/56 (48%)	31/72 (43%)	13/29 (45%)	0.43 [0.32–0.55]	0.55 [0.38–0.72]
FASH_pericardium_	44/101 (44%)	26/56 (46%)	31/72 (43%)	13/29 (45%)	0.43 [0.32–0.55]	0.55 [0.38–0.72]
FASH_pleura600ml_	22/101 (22%)	13/56 (23%)	16/72 (22%)	6/29 (21%)	0.22 [0.14–0.33]	0.79 [0.62–0.9]
Pleural effusion present, any	32/101 (32%)	19/56 (34%)	21/72 (29%)	11/29 (38%)	0.29 [0.2–0.41]	0.62 [0.44–0.77]
Pleural effusion estimated volume	346 [247–542] (N = 32)	350 [250–596] (N = 19)	350 [252–595] (N = 21)	308 [149–430] (N = 11)	…	…
Pericardial effusion ≥10 mm	5/101 (5%)	2/56 (4%)	3/72 (4%)	2/29 (7%)	0.04 [0.01–0.12]	0.93 [0.78–0.98]
Hypoechoic spleen lesions <1.5 cm present	4/101 (4%)	1/56 (2%)	1/72 (1%)	3/29 (10%)	0.01 [0–0.07]	0.9 [0.74–0.96]
Hyperechoic spleen lesions present with or without calcification	4/101 (4%)	3/56 (5%)	3/72 (4%)	1/29 (3%)	…	…
Hypoechoic liver lesions	2/101 (2%)	0/56 (0%)	0/72 (0%)	2/29 (7%)	0 [0–0.05]	0.93 [0.78–0.98]
Abdominal lymph nodes ≥1.5 cm present	9/101 (9%)	6/56 (11%)	8/72 (11%)	1/29 (3%)	0.11 [0.06–0.2]	0.97 [0.83–1]
Ascites present	6/101 (6%)	3/56 (5%)	4/72 (6%)	2/29 (7%)	0.06 [0.02–0.13]	0.93 [0.78–0.98]
Lung ultrasound						
consolidations						
Subpleural consolidations (SPC) < 1 cm present	84/101 (83%)	51/56 (91%)	60/72 (83%)	24/29 (83%)	0.83 [0.73–0.9]	0.17 [0.08–0.35]
Number of subpleural consolidations <1 cm	single: 6/84 (7) 2–5: 35/84 (42) > 5: 43/84 (51)	single: 2/56 (4) 2–5: 18/56 (32) > 5: 31/56 (55)	single: 3/60 (5) 2–5: 24 24/60 (40) > 5: 33/60 (55)	single: 3/24 (13) 2–5: 11/24 (46) > 5: 10/24 (42)	…	…
>5 SPCs <1 cm with at least one ≥5 mm	36/101 (36%)	27/56 (48%)	28/72 (39%)	8/29 (28%)	0.39 [0.29–0.51]	0.72 [0.54–0.85]
Subpleural consolidations ≥1 cm present	54/101 (53%)	39/56 (70%)	41/72 (57%)	13/29 (45%)	0.57 [0.45–0.68]	0.55 [0.38–0.72]
Any subpleural consolidation present, regardless of size?	87/101 (86%)	53/56 (95%)	63/72 (88%)	24/29 (83%)	0.88 [0.78–0.93]	0.17 [0.08–0.35]
Any consolidation in the apical regions	42/101 (42%)	31/56 (55%)	33/72 (46%)	9/29 (31%)	0.46 [0.35–0.57]	0.69 [0.51–0.83]
Any consolidations <1 cm in the apical regions	35/101 (35%)	24/56 (43%)	26/72 (36%)	9/29 (31%)	0.36 [0.26–0.48]	0.69 [0.51–0.83]
Any consolidations ≥1 cm in the apical regions	15/101 (15%)	13/56 (23%)	14/72 (19%)	1/29 (3%)	0.19 [0.12–0.3]	0.97 [0.83–1]
Other LUS findings						
Miliary pattern present	2/101 (2%)	1/56 (2%)	1/72 (1%)	1/29 (3%)	0.01 [0–0.07]	0.97 [0.83–1]
B-lines (>2) in at least 1 lung zone	81/101 (80%)	47/56 (84%)	57/72 (79%)	24/29 (83%)	0.79 [0.68–0.87]	0.17 [0.08–0.35]
LUS findings in at least … lung zones						
SPC _< 1cm_ in at least 1 lung zone	84/101 (83%)	51/56 (91%)	60/72 (83%)	24/29 (83%)	0.83 [0.73–0.9]	0.17 [0.08–0.35]
≥2 lung zones	72/101 (71%)	46/56 (82%)	54/72 (75%)	18/29 (62%)	0.75 [0.64–0.84]	0.38 [0.23–0.56]
≥3 lung zones	61/101 (60%)	39/56 (70%)	44/72 (61%)	17/29 (59%)	0.61 [0.5–0.72]	0.41 [0.26–0.59]
≥4 lung zones	52/101 (51%)	34/56 (61%)	37/72 (51%)	15/29 (52%)	0.51 [0.4–0.63]	0.48 [0.31–0.66]
≥5 lung zones	39/101 (39%)	29/56 (52%)	30/72 (42%)	9/29 (31%)	0.42 [0.31–0.53]	0.69 [0.51–0.83]
≥6 lung zones	22/101 (22%)	14/56 (25%)	14/72 (19%)	8/29 (28%)	0.19 [0.12–0.3]	0.72 [0.54–0.85]
≥7 lung zones	14/101 (14%)	9/56 (16%)	9/72 (12%)	5/29 (17%)	0.12 [0.07–0.22]	0.83 [0.65–0.92]
SPC _≥ 1cm_ in at least 1 lung zone	54/101 (53%)	39/56 (70%)	41/72 (57%)	13/29 (45%)	0.57 [0.45–0.68]	0.55 [0.38–0.72]
≥2 lung zones	29/101 (29%)	25/56 (45%)	25/72 (35%)	4/29 (14%)	0.35 [0.25–0.46]	0.86 [0.69–0.95]
≥3 lung zones	20/101 (20%)	18/56 (32%)	18/72 (25%)	2/29 (7%)	0.25 [0.16–0.36]	0.93 [0.78–0.98]
≥4 lung zones	12/101 (12%)	11/56 (20%)	11/72 (15%)	1/29 (3%)	0.15 [0.09–0.25]	0.97 [0.83–1]
B-lines in at least 1 lung zone	81/101 (80%)	47/56 (84%)	57/72 (79%)	24/29 (83%)	0.79 [0.68–0.87]	0.17 [0.08–0.35]
≥2 lung zones	57/101 (56%)	35/56 (62%)	39/72 (54%)	18/29 (62%)	0.54 [0.43–0.65]	0.38 [0.23–0.56]
≥3 lung zones	43/101 (43%)	28/56 (50%)	31/72 (43%)	12/29 (41%)	0.43 [0.32–0.55]	0.59 [0.41–0.74]
≥4 lung zones	29/101 (29%)	20/56 (36%)	20/72 (28%)	9/29 (31%)	0.28 [0.19–0.39]	0.69 [0.51–0.83]
≥5 lung zones	19/101 (19%)	15/56 (27%)	15/72 (21%)	4/29 (14%)	0.21 [0.13–0.32]	0.86 [0.6–0.95]
≥6 lung zones	13/101 (13%)	9/56 (16%)	9/72 (12%)	4/29 (14%)	0.12 [0.07–0.22]	0.86 [0.69–0.95]
Combinations of FASH and LUS						
FASH or SPC ≥ 1 cm	69/101 (68%)	45/56 (80%)	51/72 (71%)	18/29 (62%)	0.71 [0.59–0.8]	0.38 [0.23–0.56]
FASH or SPC ≥ 5 mm	84/101 (83%)	52/56 (93%)	61/72 (85%)	23/29 (79%)	0.85 [0.75–0.91]	0.21 [0.1–0.38]
Other targets						
IMNs ≥0.5 cm present	18/101 (18%)	12/56 (21%)	15/72 (21%)	3/29 (10%)	0.21 [0.13–0.32]	0.9 [0.74–0.96]
Pleural laminar thickening	14/101 (14%)	10/56 (18%)	10/72 (14%)	4/29 (14%)	…	…
Intestinal thickening in the right lower quadrant >4 mm	1/98 (1%)	0/55 (0%)	0/70 (0%)	1/28 (4%)	0 [0–0.05]	0.96 [0–82;1]
Any peritoneal thickening (parietal, visceral, omental)	2/101 (2%)	1/56 (2%)	2/72 (3%)	0/29 (0%)	0.03 [0.01–0.1]	1 [0.88–1]
Mediastinal lymph nodes seen from suprasternal view^b^	1/85 (1%)	1/44 (2%)	1/56 (2%)	0/29 (0%)	…	…
Peripheral lymph nodes present (only if clinical suspicion)	12/99 (12%)	4/55 (7%)	11/70 (16%)	1/29 (3%)	0.16 [0.09–0.26]	0.97 [0.83–1]

Denominators provided for all individuals with available data for each line.

Abbreviations: 95% CI, 95% confidence interval; CRS, composite reference standard; eMRS, extended MRS; EPTB, extrapulmonary tuberculosis; FASH, focused assessment with sonography for HIV-associated tuberculosis; IQR, interquartile range; IMN, internal mammary lymph node; LUS, lung ultrasound; MRS, microbiological reference standard; SPC, subpleural consolidation; TB, tuberculosis.

^a^In n = 31 cases of EPTB, FASH_original_ had a sensitivity of 12/31 (39%).

^b^Parasternal only negative.

### LUS

**Figure 4.**
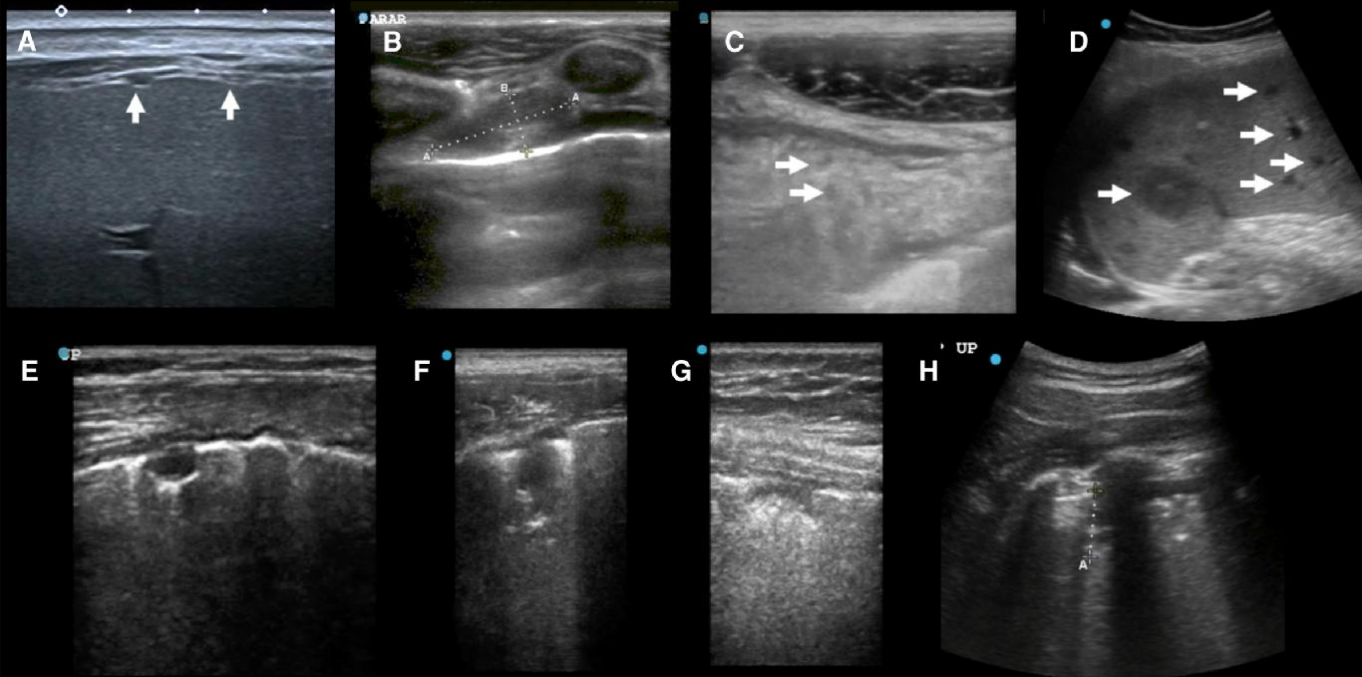
Ultrasound case studies. *A*, Female in her 40 s, DM–, HIV–, MRS+ with numerous small peritoneal nodules seen from transcostal view. *B, C*, Man in his 20s, eMRS+, internal mammary enlarged lymph node (1.9 × 0.8 cm) (*B*), and omental cake with hypoechoic inclusions (*C*, inclusions single arrows). *D*, Female in her 20s, after kidney transplant 5 y ago with unlikely TB and disseminated bartonellosis (tissue-PCR positive) with numerous small and large hypoechoic spleen lesions (maximum 2.8 cm). *E* + *F*: Female in her 40s, HIV+, post-TB with irregular treatments in the past, now MRS+ (pre-XDR), widespread SPC _< 1cm_ and SPC _≥ 1cm_ with single SPC showing oval shape (*E*), other lesions resembled shreds (*F*). *G* + *H*: Man in his 40s with large cell neuroendocrine lung carcinoma with pulmonary aspergillosis with SPC _< 1cm_ (*G*) and SPC _≥ 1cm_ (*H*, curved array). Abbreviations: CRS, composite reference standard; DM, diabetes mellitus; eMRS, extended microbiological reference standard; MRS, microbiological reference standard; SPC, subpleural consolidation; TB, tuberculosis; XDR, extensively drug resistant.

The general presence of SPC _< 1cm_ (Figure 4  *E* + *F*) in any number or zone was very common, but very nonspecific (sensitivity 83% (60/72), specificity 17% (1–24/29)). The sensitivity was increased to 91% (51/56) if limiting to PTB cases (see MRS-positive column in Supplementary Table 2). In comparison, larger SPC _≥ 1cm_ were less common but more specific (sensitivity 57% [41/72], specificity 55% [1–13/29]). Exploring the location and the number of zones affected by SPCs, we observed a high specificity, albeit lower sensitivity for SPC _≥ 1cm_ in the apical zones (sensitivity 19% [14/72], specificity 97% [1–1/29]) and also if at least 3 lung zones showed SPC _≥ 1cm_ (sensitivity 25% [18/72], specificity 93% [1–2/29]), which was comparable to “CXR suggesting likely TB” (sensitivity 27% [10/37], specificity 95% [1–1/22]; see Table 2). Miliary pattern was only seen in 1/73 (1%) TB case. Details are provided in Table 4 and additional information in Supplementary Table 2.

In unlikely TB, SPCs were mostly seen in non-TB lung infections (eg, Figure 4  *G* + *H*); a miliary pattern (n = 1) was seen in 1 participant with bronchiectasis (Table 4).

#### Combination of FASH and LUS

Exploring the performance of FASH and LUS in combination (Table 4) incrementally improved sensitivity over individual findings, while reducing the overall specificity (eg, FASH_original_ or subpleural consolidations ≥0.5 cm (any of the 2) yielded a sensitivity of 85% and a specificity of 21%).

#### Other Ultrasound Findings

IMNs were present in 15/72 (21%) TB cases and in 3/29 (10%) of unlikely TB. Pleural thickening was seen in 10/72 (14%) of TB cases and 4/29 (14%) of unlikely TB. Peritoneal thickening was seen in 2/72 (3%) TB cases (Figure 3*A*,*C*). Peripheral lymph nodes ≥1.5 cm were present in 11/70 (16%) TB cases and 1/29 (3%) unlikely TB participants.

For diabetes and HIV status subgroups, few datapoints were available because of low prevalence (Supplementary Table 3). In brief, FASH_original_ was positive in 17/37 (46%) of nondiabetic and 3/7 (43%) diabetic participants with TB. Any SPCs were seen in 7/7 (100%) of those with diabetes and 32/37 (86%) of participants without diabetes but with TB.

#### Comparator Tests

*“*CXR suggesting likely TB” had a sensitivity of 27% and a specificity of 95%, whereas “CXR suggesting possible TB” was 68% sensitive and 50% specific. CRP was 63% sensitive and 41% specific (see Table 2 & Supplementary Table 1).

Interobserver agreement was high using Cohen's kappa (0.71), and very high using the generalized linear mixed model (probability of agreement 98.8%). No adverse events were observed from testing.

## DISCUSSION

In this largest prospective TB-ultrasound study for abdominal and lung tuberculosis in a high-resource setting to date, we applied an extensive ultrasound study protocol in a triage setting for presumed TB with the purpose to explore the use case for a POCUS application. We were able to demonstrate that the accuracy of LUS and the FASH protocol as standalone tests for TB does not meet the defined targets for a standalone triage tool for TB.

FASH_original_ and variations had moderate accuracy (40% sensitivity, 55% specificity) for TB; a pleural fluid cutoff increased specificity (eg, 79% at 600 mL), whereas other FASH components did not substantially contribute to accuracy. FASH was primarily created for HIV-related TB, and heterogeneous studies showed a higher accuracy than our study cohort (pooled sensitivity of 63% and specificity of 68% for abdominal ultrasound in [8]). Comparing our mostly HIV-uninfected cohort to other studies in HIV-uninfected cohorts (eg, 36% sensitivity, 89% specificity [9] or 39% sensitivity, 70% specificity [10]) shows a similar sensitivity range. Differences in specificity likely arise because of unrepresentative control groups and differences in the study setting (eg, patients may have presented with more advanced disease in TB-endemic settings compared to our study sites, where many cases were identified through active case finding [migrant screening]). In addition, high local capacity in securing non-TB diagnoses may largely avoid clinical or false-positive TB diagnoses.

In LUS, presence of SPCs was highly nonspecific, more so than previously published (only 1 study with available specificity of 66.7% [13]). This comparison is further complicated by nonstandardized ultrasound definitions. The previously suggested entity of “subpleural nodules” (subpleural consolidations with round margins and posterior enhancement) were only seen in 6/72 (8%) TB cases (Supplementary Table 2). Most other SPC _< 1cm_ resembled irregular shaped “mini-shreds” on the linear probe (for examples, see Figure 4  *E* + *F*). Exploring SPC variations, we found accuracy comparable with the study CXR interpretation, but no LUS finding yielded accuracy above WHO target product profile targets as a standalone test. IMNs, pleural thickening, and peritoneal thickening were also mostly seen in participants with TB and may be helpful in assessing disease spread or identifying sites for sampling in EPTB. Mediastinal ultrasound has demonstrated promising data for TB screening in children [17], but reliable assessment was not possible in adults.

The complexity of diagnosis in the study population is also demonstrated by the poor performance of the comparator tests, CXR and CRP. “CXR suggesting possible TB” was 68% sensitive and 50% specific, which is less accurate than previously published accuracy data for facility-based screening (eg, 90% sensitive, 56% specific [27] or 88% sensitive, 63% specific [28], Table 2).

Reasons for the poor performance of CXR as comparator tests may be due to the TB spectrum encountered: the distribution of PTB and EPTB was skewed toward EPTB, reducing sensitivity of lung imaging. A higher prevalence of EPTB in refugees despite a low HIV prevalence was previously reported [29, 30] and may be driven by pathogen-related factors like EPTB-associated TB lineages (eg, lineage 2 predominant in Asia, lineage 4 in Europe and Africa) [31, 32]. Separately, migration stress–related host immune suppression has also been discussed as a risk factor for EPTB [29, 33]. In respect to other risk factors, we observed a high prevalence of diabetes (19%), higher than expected and related to diabetes prevalence in countries of origin overall [34, 35].

CRP at a 5 mg/L cutoff was 63% sensitive and 41% specific; see Table 2. This indicates limited applicability for screening/triage for TB in this cohort. Reasons may lie in the heterogenous origin of our cohort, which may in addition be of increased risk of migration-related immunosuppression, resulting in lower sensitivity of CRP [29].

Strengths of our study were the comprehensive ultrasound protocol, a high proportion of confirmed TB (71/73, 97%), and a stringent procedure to determine unlikely TB.

Overall, our study has some limitations. We did not reach the sample size for the cohort as a whole but exceeded target sample size for TB cases detected with the index test. For the risk group of people living with HIV, the numbers were too small to allow conclusions. Although specificity data need to be interpreted with caution, data from our Indian cohort also suggest limits in specificity [19]. The high proportions of EPTB compared to global data (see introduction) and a high proportion of diabetes may indicate a selection bias of the migrant population [34, 35]. However, the study population was largely representative for TB in Germany, with a focus in migrants (incidence foreign-born 25.1/100 000 (2022) [2]). Although our inclusion criterion was a positive WHO symptom screen [20, 36], our study population was likely further skewed by the fact that our centers operate as referral centers for TB in central and southern Germany. This also explains the high percentage of TB diagnosis in the enrolled participants. Blinding to TB status was not operationally possible in Germany in all cases (eg, where cases with a positive test result were referred to participating centers). This is a source of possible confirmation bias, but ultrasound investigators were blinded to other imaging data like CXR or computed tomography. In our partner study in India, investigators were blinded to TB status.

In conclusion, our study corroborates previous sensitivity data of FASH while showing lower specificity. It also establishes the first reliable accuracy data for LUS. Some LUS items showed moderate accuracy in the range of CXR. Ultrasound did not reach accuracy targets but its role in algorithmic approaches (eg, diagnostic scores) need to be explored. Further research is also needed to assess ultrasound performance, specifically LUS, in other risk groups (eg, children, HIV) and in a POCUS use case (ie, performed by nonradiologists) and should also consider advanced analysis approaches (eg, artificial intelligence). Furthermore, decision-making on the use of ultrasound as part of a diagnostic algorithm should also consider additional potential benefits of ultrasound in respect to delineation of extrapulmonary spread or to target diagnostic interventions.

## Supplementary Material

ofae651_Supplementary_Data

## Supplementary Data

Supplementary materials are available at *Open Forum Infectious Diseases* online. Consisting of data provided by the authors to benefit the reader, the posted materials are not copyedited and are the sole responsibility of the authors, so questions or comments should be addressed to the corresponding author.

## Notes

***Acknowledgments.*** Author Contributions (CRediT): Stefan Fabian Weber: Conceptualization, Methodology, Funding acquisition, Project administration, Investigation, Writing—original draft. Peter Wolf: Resources, Investigation. Nils Wetzstein: Resources, Project administration, Writing—review and editing. Claus Küpper-Tetzel: Resources, Writing—review and editing. Maria Vehreschild: Resources, Writing—review and editing. Isabelle Suárez: Resources, Project administration, Writing—review and editing. Jan Rybniker: Resources. Angela Klingmüller: Investigation. Tim Weber: Investigation, Writing—review and editing. Maximilian Güttlein: Investigation, Writing—review and editing. Frank Tobian: Data curation, Formal analysis. Lisa Koeppel: Formal analysis, Writing—review and editing. Julia Selena Beck: Data curation, Writing—review and editing. Rebecca Wolf: Investigation, Data curation, Writing—review and editing. Katharina Manten: Investigation, Data curation, Writing—review and editing. Stefan Zimmermann: Resources, Investigation. DJ Christopher: Methodology, writing—review and editing. Felix Herth: Resources. Sabine Bélard: Conceptualization, Writing—review and editing. Claudia Maria Denkinger: Conceptualization, Funding acquisition, Supervision, Writing—review and editing.

***Study group.*** Lisa C. Ruby: Institute of Tropical Medicine, University of Tübingen. Mary Gaeddert: University Hospital Heidelberg, Department for Infectious Disease and Tropical Medicine. Michael Weise: Thoraxklinik Heidelberg, Department for Pneumology and Critical Care Medicine. Monika Eichinger: Thoraxklinik Heidelberg, Department of Diagnostic and Interventional Radiology with Nuclear Medicine. Benjamin Blöck: University Hospital Heidelberg, Department for Infectious Disease and Tropical Medicine. Fabian Sattaf: Goethe University Frankfurt, University Hospital Frankfurt, Department 2 of Internal Medicine, Infectious Diseases, Frankfurt am Main. Anna-Lia Prey: Goethe University Frankfurt, University Hospital Frankfurt, Department 2 of Internal Medicine, Infectious Diseases, Frankfurt am Main. Alena Drummer: Goethe University Frankfurt, University Hospital Frankfurt, Department 2 of Internal Medicine, Infectious Diseases, Frankfurt am Main. Dominic Rauschning: Department I of Internal Medicine, Division of Infectious Diseases, Medical Faculty and University Hospital of Cologne, University of Cologne, Kerpener Str. 62, 50937, Cologne, Germany; and German Center for Infectious Disease Research, DZIF Partner Site Cologne. Daniel Ludwig-Bettin: Department I of Internal Medicine, Division of Infectious Diseases, Medical Faculty and University Hospital of Cologne, University of Cologne, Kerpener Str. 62, 50937, Cologne, Germany; and German Center for Infectious Disease Research, DZIF Partner Site Cologne; and Bundeswehr Central Hospital, Department IB Internal Medicine, Koblenz, Germany. Elisa Pracht: Department I of Internal Medicine, Division of Infectious Diseases, Medical Faculty and University Hospital of Cologne, University of Cologne, Kerpener Str. 62, 50937, Cologne, Germany; and German Center for Infectious Disease Research, DZIF Partner Site Cologne.

***The authors acknowledgment.*** Tom Heller for advice in POCUS protocol design (Lilongwe Lighthouse Clinic, Malawi); Sophie Schneitler for advice in POCUS protocol design (Universitätsklinikum des Saarlandes, Mikrobiologie); physicians at Thoraxklinik Heidelberg, tuberculosis ward, for study logistics, participant identification: Claudius Gottschalk, Enikö Fuchs, Raissa Liewald, Elena Langbecker, and Jasmin Jasuja.

***Financial support***. This work was supported by DZIF (Deutsches Zentrum für Infektionsforschung; German Center for Infectious Disease Research; Flexfund ID TTU 02.911). Ultrasound equipment was loaned at no cost by FujiFilm Sonosite. Research reported in this publication was supported by the National Institute of Allergy and Infectious Diseases of the US National Institutes of Health to the underlying R2D2 TB Network under award number U01AI152087.

Neither party had any role in the study design, in data collection, data analysis, data interpretation, report writing, or the decision to submit a manuscript for publication.

***Grant recipients.*** R.W., K.M., and F.T. were supported by Deutsches Zentrum für Infektionsforschung (DZIF, Grant ID Flexfund TTU 02.911). S.W. is recipient of Clinical Leave Stipend for an unrelated project by Deutsches Zentrum für Infektionsforschung (DZIF).

***Data availability***. Deidentified data will be made available upon reasonable request and provided in accordance to corresponding regulatory requirements.
