# Supplementary material for: Diagnostic Accuracy of Lung and Abdominal Ultrasound for Tuberculosis in a German Multicenter Cohort of Patients With Presumed Tuberculosis Disease
Source: Open Forum Infect Dis. 2024 Dec 9;11(12):ofae651. doi: 10.1093/ofid/ofae651 (PMC11651151; doi:10.1093/ofid/ofae651)
Supplement: ofae651_Supplementary_Data [file ofae651_supplementary_data.docx]

**Supplement**

**Manuscript: Diagnostic value of lung and abdominal ultrasound for tuberculosis in a German multi-center cohort of patients with presumed tuberculosis disease**

**Contents:**

Study protocol: DOI https://doi.org/10.11588/data/KFNN2N

SAP: DOI https://doi.org/10.11588/data/KFNN2N

Supplement Table 1 baseline cohort including MRS, eMRS, CRS

Supplement Table 2 ultrasound and variations – extended

Supplement Table 3 ultrasound stratified by HIV and diabetes status

Details Interrater Agreement

STARD 2015 checklist

| **Supplement Table 1: Baseline cohort including MRS, eMRS, CRS** | | | | | | |
| --- | --- | --- | --- | --- | --- | --- |
| **Variables in n, median, IQR, (%)** | | **All participants (n=102)** | **MRS positive (n=57)** | **eMRS positive (n=71)** | **CRS positive (n=73)** | **Unlikely TB (n=29)** |
| Age in years | | 40 [30;47] (N=102) | 38 [31;47] (N=57) | 41 [32;47] (N=71) | 41 [31;47] (N=73) | 39 [30;43] (N=29) |
| Gender male | | 80/102 (78%) | 44/57 (77%) | 55/71 (77%) | 55/73 (75%) | 25/29 (86%) |
| Body mass index (kg/m^2^) | | 22.2 [19.7;25.2] (N=101) | 21.3 [19.7;24.2] (N=56) | 22.1 [19.8;24.7] (N=70) | 22.1 [19.9;24.8] (N=72) | 22.8 [19;27.6] (N=29) |
| Origin as per WHO regions* | | 43/102 (42%) European Region  25/102 (25%) African Region  23/102 (23%) Eastern Mediterranean Region  9/102 (9%) South-East Asian Region  2/102 (2%) Western Pacific Region |  |  |  |  |
| Identifies as refugee | | 53/102 (52%)** | 26/57 (46%) | 32/71 (45%) | 34/73 (47%) | 19/29 (66%) |
| History of tobacco use | | 57/102 (56%) | 35/57 (61%) | 39/71 (55%) | 39/73 (53%) | 18/29 (62%) |
| Pack years | | 10 [6;25] (N=54) | 14 [8;24] (N=32) | 11 [7;22] (N=36) | 11 [7;22] (N=36) | 10 [6;31] (N=18) |
| History of diabetes | | 9/96 (9%) | 4/52 (8%) | 5/66 (8%) | 5/68 (7%) | 4/28 (14%) |
| Final diabetes status | | 13/69 (19%) | 6/33 (18%) | 7/43 (16%) | 7/45 (16%) | 6/24 (25%) |
| Diabetics using insulin | | 4/13 (31%) | 3/6 (50%) | 4/7 (57%) | 4/7 (57%) | 0/6 (0%) |
| History of known HIV-infection | | 6/40 (15%) | 4/25 (16%) | 5/31 (16%) | 5/32 (16%) | 1/8 (12%) |
| Patients already on ART | | 6/6 (100%) | 4/4 (100%) | 5/5 (100%) | 5/5 (100%) | 1/1 (100%) |
| Final HIV status | | 7/99 (7%) | 4/55 (7%) | 5/69 (7%) | 5/71 (7%) | 2/28 (7%) |
| History of previous COVID-19 | | 25/100 (25%) | 12/56 (21%) | 16/69 (23%) | 16/71 (23%) | 9/29 (31%) |
| History of previous COVID-19 with hospitalization | | 18/25 (72%) | 7/12 (58%) | 10/16 (62%) | 10/16 (62%) | 8/9 (89%) |
| History of previous TB disease | | 9/100 (9%) | 6/56 (11%) | 6/70 (9%) | 6/72 (8%) | 3/28 (11%) |
| History of current TB-contact | | 4/100 (4%) | 3/56 (5%) | 4/70 (6%) | 4/72 (6%) | 0/28 (0%) |
| History of previous TB-contact | | 25/100 (25%) | 13/56 (23%) | 18/70 (26%) | 18/72 (25%) | 7/28 (25%) |
| Cough | | 80/102 (78%) | 45/57 (79%) | 53/71 (75%) | 53/73 (73%) | 27/29 (93%) |
| Hemoptysis | | 16/101 (16%) | 7/57 (12%) | 7/71 (10%) | 7/73 (10%) | 9/28 (32%) |
| Night sweats | | 45/102 (44%) | 27/57 (47%) | 33/71 (46%) | 33/73 (45%) | 12/29 (41%) |
| Fever | | 32/98 (33%) | 19/54 (35%) | 21/68 (31%) | 22/70 (31%) | 10/28 (36%) |
| Weight loss | | 60/99 (61%) | 37/54 (69%) | 44/68 (65%) | 46/70 (66%) | 14/29 (48%) |
| Fatigue | | 53/101 (52%) | 33/56 (59%) | 39/70 (56%) | 40/72 (56%) | 13/29 (45%) |
| Loss of appetite | | 30/101 (30%) | 21/56 (38%) | 23/70 (33%) | 24/72 (33%) | 6/29 (21%) |
| Abdominal pain or distension | | 13/100 (13%) | 4/55 (7%) | 7/69 (10%) | 9/71 (13%) | 4/29 (14%) |
| Peripheral lymph node swelling | | 12/101 (12%) | 4/57 (7%) | 10/71 (14%) | 12/73 (16%) | 0/28 (0%) |
| HbA1c (%) | | 6 [5;6] (N=73) | 6 [5;6] (N=36) | 6 [5;6] (N=46) | 6 [5;6] (N=48) | 6 [5;6] (N=25) |
| HBA1c (%) in diabetic patients | | 7 [7;8] (N=12) | 7 [6;7] (N=5) | 7 [6;9] (N=6) | 7 [6;9] (N=6) | 7 [7;8] (N=6) |
| C-reactive protein (mg/l) | | 8 [4;30] (N=102) | 11 [4;37] (N=57) | 9 [4;33] (N=71) | 8 [4;32] (N=73) | 8 [3;25] (N=29) |
| CRP >5 mg/l** | | 63/102 (62%) | 36/57 (63%) | 44/71 (62%) | 46/73 (63%) | 17/29 (59%) |
| Hemoglobin (g/dl) | | 13 [11;14] (N=102) | 13 [11;14] (N=57) | 13 [11;14] (N=71) | 13 [11;14] (N=73) | 13 [12;14] (N=29) |
| Platelet count (/nl) | | 282 [234;364] (N=102) | 300 [245;371] (N=57) | 297 [245;375] (N=71) | 297 [245;378] (N=73) | 265 [199;308] (N=29) |
| White blood count (/nl) | | 7 [5;9] (N=102) | 7 [6;8] (N=57) | 7 [5;8] (N=71) | 7 [5;8] (N=73) | 7 [6;10] (N=29) |
| CXR suggesting likely TB | | 11/59 (19%) | 10/28 (36%) | 10/36 (28%) | 10/37 (27%) | 1/22 (5%) |
| CXR suggesting possible TB (includes likely) | | 36/59 (61%) | 24/28 (86%) | 25/36 (69%) | 25/37 (68%) | 11/22 (50%) |
| CXR suggestive of post-TB (with or without signs of active TB) | | 23/59 (39%) | 16/28 (57%) | 16/36 (44%) | 16/37 (43%) | 7/22 (32%) |
| Number of sputa investigated for TB (≥2; <2 samples) | | 98/102 (96%); 4/102 (4%) | 56/57 (98%); 1/57 (2%) | 67/71 (94%); 4/71 (6%) | 69/73 (95%); 4/73 (5%) | 29/29 (100%); n/a |
| Sputum smear status (% where done) | Negative | 60/89 (67) | 19/48 (40) | 29/58 (40) | 31/60 (52) | 29/29 (100) |
|  | Scanty | 1/89 (1) | 1/48 (2) | 1/58 (2) | 1/60 (2) |  |
|  | 1+ | 6/89 (7) | 6/48 (13) | 6/58 (10) | 6/60 (10) |  |
|  | 2+ | 6/89 (7) | 6/48 (13) | 6/58 (10) | 6/60 (10) |  |
|  | 3+ | 16/89 (18) | 16/48 (33) | 16/58 (28) | 16/60 (27) |  |
|  | Not done | 10 | 7 | 10 | 10 |  |
| Number of patients for whom BAL was investigated for TB | | 58/102 (57%) | 29/57 (51%) | 34/71 (48%) | 34/73 (47%) | 24/29 (83%) |
| Number of patients for whom non-sputum/non-BAL samples were investigated for TB | | 55/102 (54%) | 27/57 (47%) | 41/71 (58%) | 43/73 (59%) | 12/29 (41%) |
| Positive TB-PCR or culture on sputum or BAL | | 57/102 (56%) | 57/57 (100%) | 57/71 (80%) | 57/73 (78%) | 0/29 (0%) |
| Positive TB-PCR or culture on non-sputum/non-BAL sample | | 32/102 (31%) | 18/57 (32%) | 32/71 (45%) | 32/73 (44%) | 0/29 (0%) |
| PTB only  EPTB only  concurrent PTB+EPTB | | - | 42/57 (74)  0/57 (0)  15/57 (26) | 42/71 (59)  12/71 (17)  17/71 (24) | 42/73 (58);  14/73 (19);  17/73 (23) | - |

**Legend**

denominators provided for all individuals with available data for each line.

** sensitivity 63% (95% confidence interval 52-73%), specificity 41% (95% confidence interval 26-59)

IQR, interquartile range;

n, number;

MRS, microbiological reference standard;

eMRS, extended MRS;

CRS, composite reference standard;

TB, tuberculoisis;

WHO, World Health Organization;

HIV, human immunodeficiency virus;

ART, anti-retroviral therapy;

CXR, chest x-ray;

PCR, polymerase chain reaction;

BAL, broncho-alveolar lavage;

EPTB, extra-pulmonary tuberculosis

* countries of origin: Germany: 17; Eritrea: 9; Romania: 8; Ukraine: 8; India: 7; Somalia: 7; Gambia: 5; Pakistan: 5; Syria: 4; Afghanistan: 3; Morocco: 3; Algeria: 2; Georgia: 2; Kazakhstan: 2; Senegal: 2; Sudan: 2; Turkey: 2; Vietnam: 2; Bangladesh: 1; Cameroon: 1; Ethiopia: 1; France: 1; Jordania: 1; Kenya: 1; Moldavia: 1; Poland: 1; Sierra leone: 1; Slowakia: 1; Thailand: 1; Togo: 1

** <1 month: 13/53 (25%); 1-12 months: 17/53 (32%); 1-5 years: 6/53 (11%); >5 years: 17/53 (32%)

| **Supplement Table 2: Ultrasound and variations extended** | | | | | |
| --- | --- | --- | --- | --- | --- |
| **Variables in n, median, IQR, (%)** | **All participants (n=102)** | **CRS positive (n=73)** | **Unlikely TB (n=29)** | **Sensitivity (95%-CI)** | **Specificity (95%-CI)** |
| FASH | | | | | |
| FASH_original_ | 42/101 (42%) | 29/72 (40%) | 13/29 (45%) | 0.4 [0.3;0.52] | 0.55 [0.38;0.72] |
| FASH_ascites_ | 44/101 (44%) | 31/72 (43%) | 13/29 (45%) | 0.43 [0.32;0.55] | 0.55 [0.38;0.72] |
| FASH_pericardium_ | 44/101 (44%) | 31/72 (43%) | 13/29 (45%) | 0.43 [0.32;0.55] | 0.55 [0.38;0.72] |
| FASH_pleura200ml_ | 36/101 (36%) | 27/72 (38%) | 9/29 (31%) | 0.38 [0.27;0.49] | 0.69 [0.51;0.83] |
| FASH_pleura300ml_ | 32/101 (32%) | 23/72 (32%) | 9/29 (31%) | 0.32 [0.22;0.43] | 0.69 [0.51;0.83] |
| FASH_pleura400ml_ | 25/101 (25%) | 18/72 (25%) | 7/29 (24%) | 0.25 [0.16;0.36] | 0.76 [0.58;0.88] |
| FASH_pleura600ml_ | 22/101 (22%) | 16/72 (22%) | 6/29 (21%) | 0.22 [0.14;0.33] | 0.79 [0.62;0.9] |
| FASH_pleura800ml_ | 20/101 (20%) | 15/72 (21%) | 5/29 (17%) | 0.21 [0.13;0.32] | 0.83 [0.65;0.92] |
| FASH_pleura1000ml_ | 16/101 (16%) | 11/72 (15%) | 5/29 (17%) | 0.15 [0.09;0.25] | 0.83 [0.65;0.92] |
| Pleural effusion present, any | 32/101 (32%) | 21/72 (29%) | 11/29 (38%) | 0.29 [0.2;0.41] | 0.62 [0.44;0.77] |
| Pleural effusion echogenic quality | Anechoic: 19/32 (59)  mixed: 13/32 (41) | Anechoic: 10/19 (48)  mixed: 11/21 (52) | Anechoic: 9/11 (82)  mixed: 2/11 (18) |  |  |
| Pleural effusion echogenic vol | 346 [247;542] (N=32) | 350 [252;595] (N=21) | 308 [149;430] (N=11) |  |  |
| Pericardial effusion ≥4mm | 9/101 (9%) | 7/72 (10%) | 2/29 (7%) | 0.1 [0.05;0.19] | 0.93 [0.78;0.98] |
| Pericardial effusion ≥10mm | 5/101 (5%) | 3/72 (4%) | 2/29 (7%) | 0.04 [0.01;0.12] | 0.93 [0.78;0.98] |
| Pericardial effusion echogenic quality | Anechoic: 5/9 (56)  mixed: 4/9 (44) | Anechoic: 4/7 (57)  mixed: 3/7 (43) | Anechoic: 1/2 (50)  mixed: 1/2 (50) |  |  |
| Hypoechoic spleen lesions <1.5cm present | 4/101 (4%) | 1/72 (1%) | 3/29 (10%) | 0.01 [0;0.07] | 0.9 [0.74;0.96] |
| Number of hypoechoic spleen lesions | Single: 1/4 (25)  2-5: 1/4 (25)  >5: 2/4 (50) | >5: 1/1 (100) | single: 1/3 (33)  2-5: 1/3 (33)  >5: 1/3 (33) |  |  |
| Larger splenic lesions present (≥1.5cm) suggestive of macro-abscess | 1/101 (1%) | 0/72 (0%) | 1/29 (3%) |  |  |
| Hyperechoic spleen lesions present with or without calcification | 4/101 (4%) | 3/72 (4%) | 1/29 (3%) |  |  |
| Size of spleen | 11 [10;12] (N=101) | 11 [10;12] (N=72) | 11 [9;13] (N=29) |  |  |
| Hypoechoic liver lesions | 2/101 (2%) | 0/72 (0%) | 2/29 (7%) | 0 [0;0.05] | 0.93 [0.78;0.98] |
| Number of hypoechoic liver lesions | 2-5: 2/2 (100) | - | 2-5: 2/2 (100) |  |  |
| Abdominal lymph nodes ≥1.5cm present | 9/101 (9%) | 8/72 (11%) | 1/29 (3%) | 0.11 [0.06;0.2] | 0.97 [0.83;1] |
| Max. size of abdominal lymph nodes | 2 [2;2] (N=9) | 2 [2;2] (N=8) | 2 [2;2] (N=1) |  |  |
| Number of abdominal lymph nodes | single: 4/9 (44)  2-5: 2/9 (22)  >5: 3/9 (33) | single: 4/8 (50)  2-5: 1 1/8 (13)  >5: 3/8 (38) | 2-5: 1/1 (100) |  |  |
| Additional morphological pathologies seen | Necrosis: 1/2 (50)  bulking: 1/2 (50) | Necrosis: 1/2 (50)  bulking: 1/2 (50) | - |  |  |
| Ascites present | 6/101 (6%) | 4/72 (6%) | 2/29 (7%) | 0.06 [0.02;0.13] | 0.93 [0.78;0.98] |
| Ascites amount | Small: 2/6 (33)  moderate: 4/6 (67) | Moderate: 4/4 (100) | Small: 2/2 (100) |  |  |
| Ascites echogenicity | Anechoic: 5/6 (83)  mixed: 1/6 (17) | Anechoic: 3/4 (75)  mixed: 1/4 (25) | Anechoic: 2/2 (100) |  |  |
| LUNG ULTRASOUND | | | | | |
| <1cm consolidations | | | | | |
| Subpleural consolidations (SPC) <1cm present | 84/101 (83%) | 60/72 (83%) | 24/29 (83%) | 0.83 [0.73;0.9] | 0.17 [0.08;0.35] |
| SPCs with regular round/oval shape, hypoechoic echogenicity and posterior enhancement | 7/101 (7%) | 6/72 (8%) | 1/29 (3%) | 0.08 [0.04;0.17] | 0.97 [0.83;1] |
| Number of subpleural consolidations <1cm | single: 6/84 (7)  2-5: 35/84 (42)  >5: 43/84 (51) | single: 3/60 (5)  2-5: 24 24/60 (40)  >5: 33/60 (55) | single: 3/24 (13)  2-5: 11/24 (46)  >5: 10/24 (42) |  |  |
| Number of lung zones with subpleural consolidations <1cm | 4 [2;6] (N=84) | 4 [2;5] (N=60) | 4 [2;6] (N=24) |  |  |
| >5 SPCs present | 43/101 (43%) | 33/72 (46%) | 10/29 (34%) | 0.46 [0.35;0.57] | 0.66 [0.47;0.8] |
| SPCs with ≥5mm present | 68/101 (67%) | 51/72 (71%) | 17/29 (59%) | 0.72 [0.6;0.81] | 0.41 [0.26;0.59] |
| >5 SPCs with at least one ≥5mm | 36/101 (36%) | 28/72 (39%) | 8/29 (28%) | 0.39 [0.29;0.51] | 0.72 [0.54;0.85] |
| >1cm consolidations | | | | | |
| Subpleural consolidations ≥1cm present | 54/101 (53%) | 41/72 (57%) | 13/29 (45%) | 0.57 [0.45;0.68] | 0.55 [0.38;0.72] |
| Type of subpleural consolidations ≥1cm | translobar: 2/54 (4)  shred: 44/54 (81)  both: 8/54 (15) | translobar: 2/41 (5)  shred: 32/41 (78)  both: 7/41 (17) | shred: 12/13 (92)  both: 1/13 (8) |  |  |
| bronchograms visualized? | air: 34/54 (63)  air+fluid: 3/54 (6) | air: 26/41 (63)  air+fluid: 3/41 (7) | air: 8/13 (62) |  |  |
| Linear aero-bronchograms visualized? | 13/37 (35%) | 12/29 (41%) | 1/8 (12%) |  |  |
| Number of lung zones with subpleural consolidations ≥1cm | 2 [1;3] (N=54) | 2 [1;4] (N=41) | 1 [1;2] (N=13) |  |  |
| combinations of <1cm and ≥1cm | | | | | |
| any subpleural consolidation present, regardless of size? | 87/101 (86%) | 63/72 (88%) | 24/29 (83%) | 0.88 [0.78;0.93] | 0.17 [0.08;0.35] |
| max. size of consolidations, regardless of <1cm or ≥1cm | 1 [1;3] (N=86) | 2 [1;3] (N=62) | 1 [1;2] (N=24) |  |  |
| any subpleural consolidation present, size ≥5mm | 77/101 (76%) | 57/72 (79%) | 20/29 (69%) | 0.8 [0.7;0.88] | 0.31 [0.17;0.49] |
| Size of largest subpleural consolidation, categorized by small, medium, large | large: 14/87 (16)  medium: 40/87 (46)  small: 33/87 (38) | large: 13/63 (21)  medium: 28/63 (44)  small: 22/63 (35) | large: 1/24 (4)  medium: 12/24 (50)  small: 11/24 (46) |  |  |
| Location of consolidations | | | | | |
| any consolidation in the apical regions | 42/101 (42%) | 33/72 (46%) | 9/29 (31%) | 0.46 [0.35;0.57] | 0.69 [0.51;0.83] |
| any consolidations in the upper lung regions | 77/101 (76%) | 56/72 (78%) | 21/29 (72%) | 0.78 [0.67;0.86] | 0.28 [0.15;0.46] |
| any consolidations <1cm in the apical regions | 35/101 (35%) | 26/72 (36%) | 9/29 (31%) | 0.36 [0.26;0.48] | 0.69 [0.51;0.83] |
| any consolidations <1cm in the upper lung regions | 75/101 (74%) | 55/72 (76%) | 20/29 (69%) | 0.76 [0.65;0.85] | 0.31 [0.17;0.49] |
| any consolidations ≥1cm in the apical regions | 15/101 (15%) | 14/72 (19%) | 1/29 (3%) | 0.19 [0.12;0.3] | 0.97 [0.83;1] |
| any consolidations ≥1cm in the upper lung zones | 44/101 (44%) | 36/72 (50%) | 8/29 (28%) | 0.5 [0.39;0.61] | 0.72 [0.54;0.85] |
| Other LUS findings | | | | | |
| miliary pattern present | 2/101 (2%) | 1/72 (1%) | 1/29 (3%) | 0.01 [0;0.07] | 0.97 [0.83;1] |
| B-lines (>2) in at least one lung zone | 81/101 (80%) | 57/72 (79%) | 24/29 (83%) | 0.79 [0.68;0.87] | 0.17 [0.08;0.35] |
| Number of lung zones with B-lines | 3 [1;4] (N=81) | 3 [1;5] (N=57) | 2 [2;4] (N=24) |  |  |
| LUS findings depending on the number of zones affected | | | | | |
| At least 1 lung zone with subpleural consolidation <1cm | 84/101 (83%) | 60/72 (83%) | 24/29 (83%) | 0.83 [0.73;0.9] | 0.17 [0.08;0.35] |
| At least 2 lung zones with subpleural consolidation <1cm | 72/101 (71%) | 54/72 (75%) | 18/29 (62%) | 0.75 [0.64;0.84] | 0.38 [0.23;0.56] |
| At least 3 lung zones with subpleural consolidation <1cm | 61/101 (60%) | 44/72 (61%) | 17/29 (59%) | 0.61 [0.5;0.72] | 0.41 [0.26;0.59] |
| At least 4 lung zones with subpleural consolidation <1cm | 52/101 (51%) | 37/72 (51%) | 15/29 (52%) | 0.51 [0.4;0.63] | 0.48 [0.31;0.66] |
| At least 5 lung zones with subpleural consolidation <1cm | 39/101 (39%) | 30/72 (42%) | 9/29 (31%) | 0.42 [0.31;0.53] | 0.69 [0.51;0.83] |
| At least 6 lung zones with subpleural consolidation <1cm | 22/101 (22%) | 14/72 (19%) | 8/29 (28%) | 0.19 [0.12;0.3] | 0.72 [0.54;0.85] |
| At least 7 lung zones with subpleural consolidation <1cm | 14/101 (14%) | 9/72 (12%) | 5/29 (17%) | 0.12 [0.07;0.22] | 0.83 [0.65;0.92] |
| At least 8 lung zones with subpleural consolidation <1cm | 8/101 (8%) | 6/72 (8%) | 2/29 (7%) | 0.08 [0.04;0.17] | 0.93 [0.78;0.98] |
| At least 9 lung zones with subpleural consolidation <1cm | 6/101 (6%) | 4/72 (6%) | 2/29 (7%) | 0.06 [0.02;0.13] | 0.93 [0.78;0.98] |
| At least 10 lung zones with subpleural consolidation <1cm | 3/101 (3%) | 2/72 (3%) | 1/29 (3%) | 0.03 [0.01;0.1] | 0.97 [0.83;1] |
| At least 11 lung zones with subpleural consolidation <1cm | 2/101 (2%) | 1/72 (1%) | 1/29 (3%) | 0.01 [0;0.07] | 0.97 [0.83;1] |
| At least 12 lung zones with subpleural consolidation <1cm | 0/101 (0%) | 0/72 (0%) | 0/29 (0%) | 0 [0;0.05] | 1 [0.88;1] |
| At least 13 lung zones with subpleural consolidation <1cm | 0/101 (0%) | 0/72 (0%) | 0/29 (0%) | 0 [0;0.05] | 1 [0.88;1] |
| At least 14 lung zones with subpleural consolidation <1cm | 0/101 (0%) | 0/72 (0%) | 0/29 (0%) | 0 [0;0.05] | 1 [0.88;1] |
| At least 1 lung zone with subpleural consolidation ≥1cm | 54/101 (53%) | 41/72 (57%) | 13/29 (45%) | 0.57 [0.45;0.68] | 0.55 [0.38;0.72] |
| At least 2 lung zones with subpleural consolidation ≥1cm | 29/101 (29%) | 25/72 (35%) | 4/29 (14%) | 0.35 [0.25;0.46] | 0.86 [0.69;0.95] |
| At least 3 lung zones with subpleural consolidation ≥1cm | 20/101 (20%) | 18/72 (25%) | 2/29 (7%) | 0.25 [0.16;0.36] | 0.93 [0.78;0.98] |
| At least 4 lung zones with subpleural consolidation ≥1cm | 12/101 (12%) | 11/72 (15%) | 1/29 (3%) | 0.15 [0.09;0.25] | 0.97 [0.83;1] |
| At least 5 lung zones with subpleural consolidation ≥1cm | 4/101 (4%) | 4/72 (6%) | 0/29 (0%) | 0.06 [0.02;0.13] | 1 [0.88;1] |
| At least 6 lung zones with subpleural consolidation ≥1cm | 3/101 (3%) | 3/72 (4%) | 0/29 (0%) | 0.04 [0.01;0.12] | 1 [0.88;1] |
| At least 7 lung zones with subpleural consolidation ≥1cm | 1/101 (1%) | 1/72 (1%) | 0/29 (0%) | 0.01 [0;0.07] | 1 [0.88;1] |
| At least 8 lung zones with subpleural consolidation ≥1cm | 1/101 (1%) | 1/72 (1%) | 0/29 (0%) | 0.01 [0;0.07] | 1 [0.88;1] |
| At least 9 lung zones with subpleural consolidation ≥1cm | 1/101 (1%) | 1/72 (1%) | 0/29 (0%) | 0.01 [0;0.07] | 1 [0.88;1] |
| At least 10 lung zones with subpleural consolidation ≥1cm | 0/101 (0%) | 0/72 (0%) | 0/29 (0%) | 0 [0;0.05] | 1 [0.88;1] |
| At least 11 lung zones with subpleural consolidation ≥1cm | 0/101 (0%) | 0/72 (0%) | 0/29 (0%) | 0 [0;0.05] | 1 [0.88;1] |
| At least 12 lung zones with subpleural consolidation ≥1cm | 0/101 (0%) | 0/72 (0%) | 0/29 (0%) | 0 [0;0.05] | 1 [0.88;1] |
| At least 13 lung zones with subpleural consolidation ≥1cm | 0/101 (0%) | 0/72 (0%) | 0/29 (0%) | 0 [0;0.05] | 1 [0.88;1] |
| At least 14 lung zones with subpleural consolidation ≥1cm | 0/101 (0%) | 0/72 (0%) | 0/29 (0%) | 0 [0;0.05] | 1 [0.88;1] |
| At least 1 lung zone with B-lines | 81/101 (80%) | 57/72 (79%) | 24/29 (83%) | 0.79 [0.68;0.87] | 0.17 [0.08;0.35] |
| At least 2 lung zones with B-lines | 57/101 (56%) | 39/72 (54%) | 18/29 (62%) | 0.54 [0.43;0.65] | 0.38 [0.23;0.56] |
| At least 3 lung zones with B-lines | 43/101 (43%) | 31/72 (43%) | 12/29 (41%) | 0.43 [0.32;0.55] | 0.59 [0.41;0.74] |
| At least 4 lung zones with B-lines | 29/101 (29%) | 20/72 (28%) | 9/29 (31%) | 0.28 [0.19;0.39] | 0.69 [0.51;0.83] |
| At least 5 lung zones with B-lines | 19/101 (19%) | 15/72 (21%) | 4/29 (14%) | 0.21 [0.13;0.32] | 0.86 [0.69;0.95] |
| At least 6 lung zones with B-lines | 13/101 (13%) | 9/72 (12%) | 4/29 (14%) | 0.12 [0.07;0.22] | 0.86 [0.69;0.95] |
| At least 7 lung zones with B-lines | 8/101 (8%) | 5/72 (7%) | 3/29 (10%) | 0.07 [0.03;0.15] | 0.9 [0.74;0.96] |
| At least 8 lung zones with B-lines | 8/101 (8%) | 5/72 (7%) | 3/29 (10%) | 0.07 [0.03;0.15] | 0.9 [0.74;0.96] |
| At least 9 lung zones with B-lines | 7/101 (7%) | 5/72 (7%) | 2/29 (7%) | 0.07 [0.03;0.15] | 0.93 [0.78;0.98] |
| At least 10 lung zones with B-lines | 3/101 (3%) | 1/72 (1%) | 2/29 (7%) | 0.01 [0;0.07] | 0.93 [0.78;0.98] |
| At least 11 lung zones with B-lines | 3/101 (3%) | 1/72 (1%) | 2/29 (7%) | 0.01 [0;0.07] | 0.93 [0.78;0.98] |
| At least 12 lung zones with B-lines | 2/101 (2%) | 1/72 (1%) | 1/29 (3%) | 0.01 [0;0.07] | 0.97 [0.83;1] |
| At least 13 lung zones with B-lines | 1/101 (1%) | 0/72 (0%) | 1/29 (3%) | 0 [0;0.05] | 0.97 [0.83;1] |
| At least 14 lung zones with B-lines | 1/101 (1%) | 0/72 (0%) | 1/29 (3%) | 0 [0;0.05] | 0.97 [0.83;1] |
| OTHER US TARGETS | | | | | |
| Internal mammary lymph nodes (IMNs) ≥0.5cm present | 18/101 (18%) | 15/72 (21%) | 3/29 (10%) | 0.21 [0.13;0.32] | 0.9 [0.74;0.96] |
| max. size of IMNs | 11 [6;17] (N=18) | 11 [7;18] (N=15) | 6 [6;10] (N=3) |  |  |
| side of IMNs | Unilateral: 13/18 (72)  Bilateral: 5/18 (28) | Unilateral: 11/15 (73)  Bilateral: 4/15 (27) | Unilateral: 2/3 (67)  bilateral: 1/3 (33) |  |  |
| Additional IMN pathology | necrosis: 2/18 (11)  bulking: 1/18 (6) | necrosis: 2/15 (13)  bulking: 1/15 (7) | - |  |  |
| Pleural Nodules | 4/101 (4%) | 4/72 (6%) | 0/29 (0%) |  |  |
| Pleural laminar thickening | 14/101 (14%) | 10/72 (14%) | 4/29 (14%) |  |  |
| Pleural nodules or laminar thickening | 14/101 (14%) | 10/72 (14%) | 4/29 (14%) |  |  |
| diameter of pleural nodule (mm) | 6 [6;8] (N=4) | 6 [6;8] (N=4) | - |  |  |
| laminar pleural thickening, maximum thickness (mm)? | 3 [3;4] (N=13) | 3 [3;4] (N=9) | 3 [3;3] (N=4) |  |  |
| Intestinal thickening in the right lower quadrant >4mm | 1/98 (1%) | 0/70 (0%) | 1/28 (4%) | 0 [0;0.05] | 0.96 [0.82;1] |
| max. size of intestinal thickening | 5 [5;5] (N=1) | NA [NA;NA] (N=0) | 5 [5;5] (N=1) |  |  |
| Any peritoneal thickening (parietal, visceral, omental) | 2/101 (2%) | 2/72 (3%) | 0/29 (0%) | 0.03 [0.01;0.1] | 1 [0.88;1] |
| Mediastinal lymph nodes seen from suprasternal view [parasternal only negative] | 1/85 (1%) | 1/56 (2%) | 0/29 (0%) |  |  |
| Peripheral lymph nodes present (only if clinical suspicion) | 12/99 (12%) | 11/70 (16%) | 1/29 (3%) | 0.16 [0.09;0.26] | 0.97 [0.83;1] |
| **Legend**  denominators provided for all individuals with available data for each line.  ** sensitivity 63% (95% confidence interval 52-73%), specificity 41% (95% confidence interval 26-59)  IQR, interquartile range;  n, number;  MRS, microbiological reference standard;  eMRS, extended MRS;  CRS, composite reference standard;  TB, tuberculosis;  FASH, focused assessment with sonography for HIV-associated tuberculosis, subscript definitions: ascites (FASH positive also when ascites is detected), pericardium (FASH positive also with lower pericardial effusion cut-off of 4mm), pleura (FASH only positive if minimum pleural fluid amount is exceeded), see also Table 1 main manuscript;  SPC, subpleural consolidation;  LUS, lung ultrasound;  IMN, internal mammary lymph node | | | | | |

**Supplement Table 3: ultrasound stratified by HIV and diabetes status**

|  | DM-, all (n=56) | DM-, CRS+ (n=38) | DM-, unlikely TB (n=18) | DM+, all (n=13) | DM+, CRS+ (n=7) | DM+, unlikely TB (n=6) | HIV-, all (n=92) | HIV-, CRS+ (n=66) | HIV-, unlikely TB (n=26) | HIV+, all (n=7) | HIV+, CRS+ (n=5) | HIV+, unlikely TB (n=2) |
| --- | --- | --- | --- | --- | --- | --- | --- | --- | --- | --- | --- | --- |
| DIAGNOSTICS | | | | | | | | | | | | |
| CXR suggestive of active TB | 6/37 (16%) | 5/22 (23%) | 1/15 (7%) | 2/8 (25%) | 2/6 (33%) | 0/2 (0%) | 8/52 (15%) | 7/33 (21%) | 1/19 (5%) | 3/5 (60%) | 3/3 (100%) | 0/2 (0%) |
| CXR suggestive or consistent with active TB | 23/37 (62%) | 14/22 (64%) | 9/15 (60%) | 5/8 (62%) | 5/6 (83%) | 0/2 (0%) | 30/52 (58%) | 21/33 (64%) | 9/19 (47%) | 4/5 (80%) | 3/3 (100%) | 1/2 (50%) |
| Positive TB-PCR on sputum or BAL | 24/56 (43%) | 24/38 (63%) | 0/18 (0%) | 6/13 (46%) | 6/7 (86%) | 0/6 (0%) | 43/92 (47%) | 43/66 (65%) | 0/26 (0%) | 4/7 (57%) | 4/5 (80%) | 0/2 (0%) |
| Positive TB-culture on sputum or BAL | 25/56 (45%) | 25/38 (66%) | 0/18 (0%) | 6/13 (46%) | 6/7 (86%) | 0/6 (0%) | 48/92 (52%) | 48/66 (73%) | 0/26 (0%) | 4/7 (57%) | 4/5 (80%) | 0/2 (0%) |
| Positive TB-PCR on EPTB sample | 12/56 (21%) | 12/38 (32%) | 0/18 (0%) | 0/13 (0%) | 0/7 (0%) | 0/6 (0%) | 19/92 (21%) | 19/66 (29%) | 0/26 (0%) | 1/7 (14%) | 1/5 (20%) | 0/2 (0%) |
| Positive TB-culture on EPTB sample | 12/56 (21%) | 12/38 (32%) | 0/18 (0%) | 2/13 (15%) | 2/7 (29%) | 0/6 (0%) | 25/92 (27%) | 25/66 (38%) | 0/26 (0%) | 1/7 (14%) | 1/5 (20%) | 0/2 (0%) |
| PTB only  EPTB only  PTB+EPTB | PTB: 19/34 (56)  EPTB: 11/34 (32)  PTB+EPTB: 8/34 (24) | | | PTB: 6/7 (86) PTB+EPTB: 1/7 (14) | | | PTB: 38/66 (58)  EPTB: 14/66 (21)  PTB+EPTB: 14/66 (21) | | | PTB: 3/5 (60)  EPTB: 1/5 (20)  PTB+EPTB: 1/5 (20) | | |
| FASH | | | | | | | | | | | | |
| FASH (original) positive | 24/55 (44%) | 17/37 (46%) | 7/18 (39%) | 7/13 (54%) | 3/7 (43%) | 4/6 (67%) | 37/91 (41%) | 25/65 (38%) | 12/26 (46%) | 4/7 (57%) | 3/5 (60%) | 1/2 (50%) |
| Pleural effusion present, any | 19/55 (35%) | 12/37 (32%) | 7/18 (39%) | 6/13 (46%) | 3/7 (43%) | 3/6 (50%) | 29/91 (32%) | 19/65 (29%) | 10/26 (38%) | 2/7 (29%) | 1/5 (20%) | 1/2 (50%) |
| Pericardial effusion ≥10mm | 4/55 (7%) | 3/37 (8%) | 1/18 (6%) | 1/13 (8%) | 0/7 (0%) | 1/6 (17%) | 5/91 (5%) | 3/65 (5%) | 2/26 (8%) | 0/7 (0%) | 0/5 (0%) | 0/2 (0%) |
| Hypoechoic spleen lesions <1.5cm present | 3/55 (5%) | 1/37 (3%) | 2/18 (11%) | 0/13 (0%) | 0/7 (0%) | 0/6 (0%) | 3/91 (3%) | 0/65 (0%) | 3/26 (12%) | 1/7 (14%) | 1/5 (20%) | 0/2 (0%) |
| Hypoechoic liver lesions | 1/55 (2%) | 0/37 (0%) | 1/18 (6%) | 1/13 (8%) | 0/7 (0%) | 1/6 (17%) | 2/91 (2%) | 0/65 (0%) | 2/26 (8%) | 0/7 (0%) | 0/5 (0%) | 0/2 (0%) |
| Abdominal lymph nodes ≥1.5cm present | 6/55 (11%) | 5/37 (14%) | 1/18 (6%) | 0/13 (0%) | 0/7 (0%) | 0/6 (0%) | 6/91 (7%) | 5/65 (8%) | 1/26 (4%) | 3/7 (43%) | 3/5 (60%) | 0/2 (0%) |
| Ascites present | 4/55 (7%) | 2/37 (5%) | 2/18 (11%) | 1/13 (8%) | 1/7 (14%) | 0/6 (0%) | 6/91 (7%) | 4/65 (6%) | 2/26 (8%) | 0/7 (0%) | 0/5 (0%) | 0/2 (0%) |
| LUNG ULTRASOUND | | | | | | | | | | | | |
| Subpleural consolidations (SPC) <1cm present | 46/55 (84%) | 31/37 (84%) | 15/18 (83%) | 11/13 (85%) | 6/7 (86%) | 5/6 (83%) | 76/91 (84%) | 54/65 (83%) | 22/26 (85%) | 6/7 (86%) | 5/5 (100%) | 1/2 (50%) |
| Subpleural consolidations ≥1cm present | 30/55 (55%) | 21/37 (57%) | 9/18 (50%) | 9/13 (69%) | 6/7 (86%) | 3/6 (50%) | 49/91 (54%) | 37/65 (57%) | 12/26 (46%) | 4/7 (57%) | 3/5 (60%) | 1/2 (50%) |
| any subpleural consolidation present, regardless of size? | 47/55 (85%) | 32/37 (86%) | 15/18 (83%) | 12/13 (92%) | 7/7 (100%) | 5/6 (83%) | 78/91 (86%) | 56/65 (86%) | 22/26 (85%) | 6/7 (86%) | 5/5 (100%) | 1/2 (50%) |
| miliary pattern present | 1/55 (2%) | 1/37 (3%) | 0/18 (0%) | 1/13 (8%) | 0/7 (0%) | 1/6 (17%) | 2/91 (2%) | 1/65 (2%) | 1/26 (4%) | 0/7 (0%) | 0/5 (0%) | 0/2 (0%) |
| B-lines (>2) in at least one lung zone | 43/55 (78%) | 29/37 (78%) | 14/18 (78%) | 12/13 (92%) | 6/7 (86%) | 6/6 (100%) | 72/91 (79%) | 50/65 (77%) | 22/26 (85%) | 6/7 (86%) | 5/5 (100%) | 1/2 (50%) |
| Other ultrasound targets | | | | | | | | | | | | |
| Internal mammary lymph nodes (IMNs) ≥0.5cm present | 13/55 (24%) | 10/37 (27%) | 3/18 (17%) | 2/13 (15%) | 2/7 (29%) | 0/6 (0%) | 17/91 (19%) | 14/65 (22%) | 3/26 (12%) | 1/7 (14%) | 1/5 (20%) | 0/2 (0%) |
| Pleural nodules or laminar thickening | 9/55 (16%) | 5/37 (14%) | 4/18 (22%) | 1/13 (8%) | 1/7 (14%) | 0/6 (0%) | 11/91 (12%) | 8/65 (12%) | 3/26 (12%) | 2/7 (29%) | 2/5 (40%) | 0/2 (0%) |
| Intestinal thickening in the right lower quadrant >4mm | 1/53 (2%) | 0/36 (0%) | 1/17 (6%) | 0/13 (0%) | 0/7 (0%) | 0/6 (0%) | 1/88 (1%) | 0/63 (0%) | 1/25 (4%) | 0/7 (0%) | 0/5 (0%) | 0/2 (0%) |
| Any peritoneal thickening (parietal, visceral, omental) | 2/55 (4%) | 2/37 (5%) | 0/18 (0%) | 0/13 (0%) | 0/7 (0%) | 0/6 (0%) | 2/91 (2%) | 2/65 (3%) | 0/26 (0%) | 0/7 (0%) | 0/5 (0%) | 0/2 (0%) |
| Mediastinal lymph nodes seen from suprasternal view [parasternal only negative] | 1/53 (2%) | 1/35 (3%) | 0/18 (0%) | 0/13 (0%) | 0/7 (0%) | 0/6 (0%) | 1/76 (1%) | 1/50 (2%) | 0/26 (0%) | 0/7 (0%) | 0/5 (0%) | 0/2 (0%) |
| Peripheral lymph nodes present (only if clinical suspicion) | 8/55 (15%) | 8/37 (22%) | 0/18 (0%) | 1/13 (8%) | 0/7 (0%) | 1/6 (17%) | 9/89 (10%) | 8/63 (13%) | 1/26 (4%) | 2/7 (29%) | 2/5 (40%) | 0/2 (0%) |
| **Legend**  DM, diabetes mellitus;  n, number;  HIV, human immunodeficiency virus;  CRS, composite reference standard;  CXR, chest x-ray;  TB, tuberculosis;  PCR, polymerase chain reaction;  BAL, broncho-alveolar lavage;  EPTB, extra-pulmonary tuberculosis;  PTB, pulmonary tuberculosis;  FASH, focused assessment with sonography for HIV-associated tuberculosis;  SPC, subpleural consolidations;  IMN, internal mammary lymph nodes | | | | | | | | | | | | |

Inter rater agreement

Interobserver agreement calculations (see methods) yielded an overall Cohen’s kappa of 0.71, and more specifically for rater 1 and 2: 0.65, raters 1 and 3: 0.79, and raters 2 and 3: 0.72. From the generalized linear mixed we inferred the probability of agreement to be 98.8%, which strongly supports coherence in rater decisions.

**STARD 2015**

|  | **Section & Topic** | **No** | **Item** | **Reported on page #** |
| --- | --- | --- | --- | --- |
|  |  |  |  |  |
|  | **TITLE OR ABSTRACT** |  |  |  |
|  |  | **1** | Identification as a study of diagnostic accuracy using at least one measure of accuracy  (such as sensitivity, specificity, predictive values, or AUC) | 1 |
|  | **ABSTRACT** |  |  |  |
|  |  | **2** | Structured summary of study design, methods, results, and conclusions  (for specific guidance, see STARD for Abstracts) | 2 |
|  | **INTRODUCTION** |  |  |  |
|  |  | **3** | Scientific and clinical background, including the intended use and clinical role of the index test | 4 |
|  |  | **4** | Study objectives and hypotheses | 4 |
|  | **METHODS** |  |  |  |
|  | *Study design* | **5** | Whether data collection was planned before the index test and reference standard  were performed (prospective study) or after (retrospective study) | 5 |
|  | *Participants* | **6** | Eligibility criteria | 5 |
|  |  | **7** | On what basis potentially eligible participants were identified  (such as symptoms, results from previous tests, inclusion in registry) | 5 |
|  |  | **8** | Where and when potentially eligible participants were identified (setting, location and dates) | 5, 9 |
|  |  | **9** | Whether participants formed a consecutive, random or convenience series | 5 |
|  | *Test methods* | **10a** | Index test, in sufficient detail to allow replication | 6, 7 |
|  |  | **10b** | Reference standard, in sufficient detail to allow replication | 7 |
|  |  | **11** | Rationale for choosing the reference standard (if alternatives exist) | 7 |
|  |  | **12a** | Definition of and rationale for test positivity cut-offs or result categories  of the index test, distinguishing pre-specified from exploratory | 6, 7, Table 1 |
|  |  | **12b** | Definition of and rationale for test positivity cut-offs or result categories  of the reference standard, distinguishing pre-specified from exploratory | 7 |
|  |  | **13a** | Whether clinical information and reference standard results were available  to the performers/readers of the index test | 6 |
|  |  | **13b** | Whether clinical information and index test results were available  to the assessors of the reference standard | 7 |
|  | *Analysis* | **14** | Methods for estimating or comparing measures of diagnostic accuracy | 8 |
|  |  | **15** | How indeterminate index test or reference standard results were handled | 6, 7 |
|  |  | **16** | How missing data on the index test and reference standard were handled | 7 |
|  |  | **17** | Any analyses of variability in diagnostic accuracy, distinguishing pre-specified from exploratory | 6, Table 1, Table 3 |
|  |  | **18** | Intended sample size and how it was determined | 8 |
|  | **RESULTS** |  |  |  |
|  | *Participants* | **19** | Flow of participants, using a diagram | Fig. 2 |
|  |  | **20** | Baseline demographic and clinical characteristics of participants | Table 2 |
|  |  | **21a** | Distribution of severity of disease in those with the target condition | Table 2 |
|  |  | **21b** | Distribution of alternative diagnoses in those without the target condition | 8, Table 3 |
|  |  | **22** | Time interval and any clinical interventions between index test and reference standard | 6 |
|  | *Test results* | **23** | Cross tabulation of the index test results (or their distribution)  by the results of the reference standard | Table 4 |
|  |  | **24** | Estimates of diagnostic accuracy and their precision (such as 95% confidence intervals) | Table 4 |
|  |  | **25** | Any adverse events from performing the index test or the reference standard |  |
|  | **DISCUSSION** |  |  |  |
|  |  | **26** | Study limitations, including sources of potential bias, statistical uncertainty, and generalisability | 12, 13 |
|  |  | **27** | Implications for practice, including the intended use and clinical role of the index test | 11, 12 |
|  | **OTHER INFORMATION** |  |  |  |
|  |  | **28** | Registration number and name of registry | Methods, 4 |
|  |  | **29** | Where the full study protocol can be accessed | Supplement |
|  |  | **30** | Sources of funding and other support; role of funders | 14 |
|  |  |  |  |  |
